# Supplementary material for: Identification of unusual oxysterols biosynthesised in human pregnancy by charge-tagging and liquid chromatography - mass spectrometry
Source: Front Endocrinol (Lausanne). 2022 Nov 10;13:1031013. doi: 10.3389/fendo.2022.1031013 (PMC9685423; doi:10.3389/fendo.2022.1031013)

Supplementary Material

1 Supplemental Materials and Methods

1.1 LC-MS Mobile Phase and Gradient

LC was performed on an Ultimate 3000 UHPLC system (Dionex, now Thermo Fisher Scientific, Hemel Hempstead, UK) using a Hypersil GOLD revered phase column (1.9 µm particle size, 50 × 2.1 mm, Thermo Fisher). Mobile phase A consisted of 33.3% methanol, 16.7% acetonitrile and 0.1% formic acid. Mobile phase B consisted of 63.3% methanol, 31.7% acetonitrile and 0.1% formic acid. The 17 min chromatographic run started at 20% B for 1 min, before increasing the proportion of B to 80% over 7 minutes, and maintaining this for a further 5 min. The proportion of B was returned to 20% over 6 s and re-equilibration was for 3 min, 54 s to give a total run time of 17 min. To separate closely eluting oxysterols, the usual 17 min gradient was extended to 37 min. The mobile phase composition was initially at 20% B for 10 min, changed to 50% B over the next 10 min, maintained at this composition for 6 min, and then changed 80% B over the next 3 min. The mobile phase composition was held at 80% B for a further 3 min before returning to 20% B in 6 s and reconditioning the column for a further 4 min 54 s.

The flow rate was 200 µL/min and the eluent was directed to the atmospheric pressure ionization (API) source of an Orbitrap Elite MS (Thermo Fisher Scientific). The Orbitrap was calibrated externally, and the mass accuracy was better than 5 ppm.

1.2 Scan Events on the Orbitrap Elite MS

The scan events were as follows. For each LC-MS(MS^n) injection the first scan event consisted of a Fourier Transform (FT)-MS scan in the Orbitrap at 120,000 resolution (full width at half-maximum height; FWHM) at *m/z* 400, simultaneous to which sequential MS^3 scan events were carried out in the linear ion-trap with normalised collision energies of 30 for MS^2 and 35 for MS^3 (instrument settings).

Injection (run time)	Orbitrap *m/z*	LIT MS^3	LIT MS^3	LIT MS^3	LIT MS^3	LIT MS^3
1 (17 min)	400-610	522.3→443.3→	527.4→443.3→	532.4→453.4→	537.4→453.4→	
2 (17 min)	400-610	534.4→455.4→	539.4→455.4→	550.4→471.4→	555.4→471.4→	
3 (17 min)	400-610	546.4→467.4→	551.4→467.3→	564.4→485.3→	569.4→485.3→	
4 (17 min)	400-610	546.5→462.4→	548.4→469.3→	553.4→469.3→	580.4→501.3→	585.4→501.3→
5 (17 min)	400-610	562.4→483.3→	566.4→487.4→	567.4→483.3→	571.4→487.4→	
6 (17 min)	400-610	537.4→435.3→	543.5→441.4→	556.4→477.4→	561.5→477.4→	
7 (17 min)	300-800	614.4→535.3→	619.4→535.3→	710.4→631.4→	715.5→631.4→	
8 (17 min)	400-610	506.3→427.3→	511.4→427.3→	578.4→499.3→	583.4→499.3→	

9 (37 min)	400-610	539.4→455.4→	546.5→462.4→	550.4→471.4→	555.4→471.4→	
10 (17 min)	400-610	540.4→461.4→	541.5→462.4→	545.5→461.4→	546.5→462.4→	

2 Supplemental Figures

Supplemental Figure S1. (A) Schematic illustration of the EADSA process exemplified by 20R,22R-diHC and 7α,(25R)26-dihydroxycholesterol (7α,26-diHC) in fraction A and 7α,(25R)26-dihydroxycholest-4-en-3-one (7α,26-diHCO) in fraction B. (B) *Z* (*syn*) and *E* (*anti*) conformers of the GP-derivative of 22R-HC. The insets show minimum energy structures calculated by Chem 3DPro (Perkin Elmer), lone pairs are in pink. Note the *Z* conformer is of lower energy than the *E* conformer, thus the lower energy structure of both becomes the *Z* conformer, as conformers are by definition interconvertible. (C) Characteristic MS^3 fragmentation of GP-derivatised 3β-hydroxy-5-ene sterols. The top box shows sterol numbering and fragmentation nomenclature (1). An asterisk indicates that the pyridine ring has been lost. A prime to the left of the fragment describing letter, e.g. '*e, indicates that homolytic fragmentation proceeds with loss of an additional hydrogen atom from the ion. A prime to the right, e.g. *e', indicates that homolytic fragmentation proceeds with the addition of a hydrogen atom to the fragment ion. The central box shows the major ring-fragment ions. The lower box shows the changed pattern of ring fragment ions when a hydroxy group is present on C-7. (D) GP-derivatised 24S-HC gives a major side-chain fragment ion at *m/z* 353.3 ('*f) and a minor fragment ion at 325.2 ('*e). See Supplemental Figure S3 for MS^3 spectrum. Coloured arrows suggest possible mechanisms and the minimum energy structure, showing lone-pairs in pink, of the intermediate ion at *m/z* 437.4 is shown in the inset. Square brackets indicate different excited states.

Supplemental Figure S2. Suggested mechanisms for the formation of side-chain fragment ions in GP-derivatised 20-, and 22-hydroxysterols. (A) 22R-HC. (B) 20S-HC. (C-D) 20R,22R-diHC. (E) 22S,23-diHC. (F) 20R,22R,23-triHC. (G) 20R,22R,24-triHC. (H-I) 20R,22R,26-triHC. (J-L) 3β,20R,22R-triHCA. (M-O) 3β,20R,22R-triH-Δ^{24}-CA. Square brackets indicate different tautomers or excited states. Coloured arrows suggest possible mechanisms. Minimum energy structures, showing lone pairs in pink, of key intermediate ions are shown in insets. Major side-chain fragmentations are driven by the stability of ketene, ketone or enol products. MS^3 spectra can be found in Figures 2B (22R-HC), 2F (20S-HC), 3B (20R,22R-diHC), 3C (22,23-diHC), 3G (20R,22R,26-triHC), 3H (20R,22R,23-triHC & 20R,22R,24-triHC), 4B (3β,20R,22R-triHCA) and 4F (3β,20R,22R-triH-Δ^{24}-CA).

Supplemental Figure S3. Partial resolution by LC-MS(MS^n) of GP-derivatised 20S-HC from 24S-HC and other hydroxycholesterols in placental samples. (A) RIC using the extended chromatographic gradient (37 min) for 20S-HC, 24S-HC and 26-HC (*m/z* 539.4368 ± 5 ppm, upper panel). MRM-like chromatograms targeting 22S-HC ($[M]^+$→$[M-Py]^+$→327, 2nd panel), 24S-HC ($[M]^+$→$[M-Py]^+$→353, 3rd panel) and [$^2H_7$]24R/S-HC ($[M]^+$→$[M-Py]^+$→353, bottom panel). Note GP derivatives each give *syn* and *anti* conformers resulting in twin peaks. (B-C) MS^3 ($[M]^+$→$[M-Py]^+$→) spectra of 24S-HC (upper panels) and [$^2H_7$]24S-HC (lower panels). Note deuterated compounds elute slightly earlier than their hydrogen equivalents. (D) MS^3 ($[M]^+$→$[M-Py]^+$→) spectra of the 26-HC. (E) RIC using the 17 min chromatographic gradient for monohydroxycholesterols (*m/z* 539.4368 ± 5 ppm, upper panel) and TIC generated by MS^3 ($[M]^+$→$[M-Py]^+$→, lower panel). MS^3 ($[M]^+$→$[M-Py]^+$→) spectra of (F) 7β-HC and (G) 7α-HC. Chromatograms using the 17 min gradient were aligned to the 26-HC

peak in the NIST SRM 1950 plasma as described for Figure 2. Spectra of authentic standards can be found in (2).

Supplemental Figure S4. LC-MS(MS^n) of GP-derivatised cholestenoic acids in placenta. (A) RIC (*m/z* 553.4161 ± 5 ppm, upper panel) corresponding to the $[M]^+$ ion of 3β-HCA and TIC generated by MS^3 ($[M]^+$→$[M\text{-}Py]^+$→, lower panel). (B) MS^3 ($[M]^+$→$[M\text{-}Py]^+$→) spectrum of 3β-HCA. Chromatograms using the 17 min gradient were aligned to the 26-HC peak in the NIST SRM 1950 plasma as described for Figure 2. Spectra of authentic standards can be found in (2).

Supplemental Figure S5. LC-MS(MS^n) analysis of GP-derivatised trihydroxycholestadienoic acids found in cord plasma and amniotic fluid. (A) RIC (*m/z* 583.3902 ± 5 ppm, upper panel) appropriate to 3β,20R,22R-triH-Δ^{24}-CA and MRM-like chromatograms targeting this acid, $[M]^+$→$[M\text{-}Py]^+$→327 (centre panel) and $[M]^+$→$[M\text{-}Py]^+$→353 (lower panel) from cord plasma. (B) MS^3 ($[M]^+$→$[M\text{-}Py]^+$→) spectrum postulated to correspond to 3β,20R,22R-triH-Δ^{24}-CA in cord plasma. (C) RIC (*m/z* 583.3902 ± 5 ppm, upper panel) appropriate to 3β,20R,22R-triH-Δ^{24}-CA and MRM-like chromatogram targeting this acid $[M]^+$→$[M\text{-}Py]^+$→327 (lower panel) from amniotic fluid. (D) MS^3 ($[M]^+$→$[M\text{-}Py]^+$→) spectrum postulated to correspond to 3β,20R,22R-triH-Δ^{24}-CA in amniotic fluid.

1. Karu, K., M. Hornshaw, G. Woffendin, K. Bodin, M. Hamberg, G. Alvelius, J. Sjovall, J. Turton, Y. Wang, and W. J. Griffiths. 2007. Liquid chromatography-mass spectrometry utilizing multi-stage fragmentation for the identification of oxysterols. *J Lipid Res* **48**: 976-987.

2. Yutuc, E., A. L. Dickson, M. Pacciarini, L. Griffiths, P. R. S. Baker, L. Connell, A. Öhman, L. Forsgren, M. Trupp, S. Vilarinho, Y. Khalil, P. T. Clayton, S. Sari, B. Dalgic, P. Höflinger, L. Schöls, W. J. Griffiths, and Y. Wang. 2021. Deep mining of oxysterols and cholestenoic acids in human plasma and cerebrospinal fluid: Quantification using isotope dilution mass spectrometry. *Anal Chim Acta* **1154**: 338259.

Figure S1A

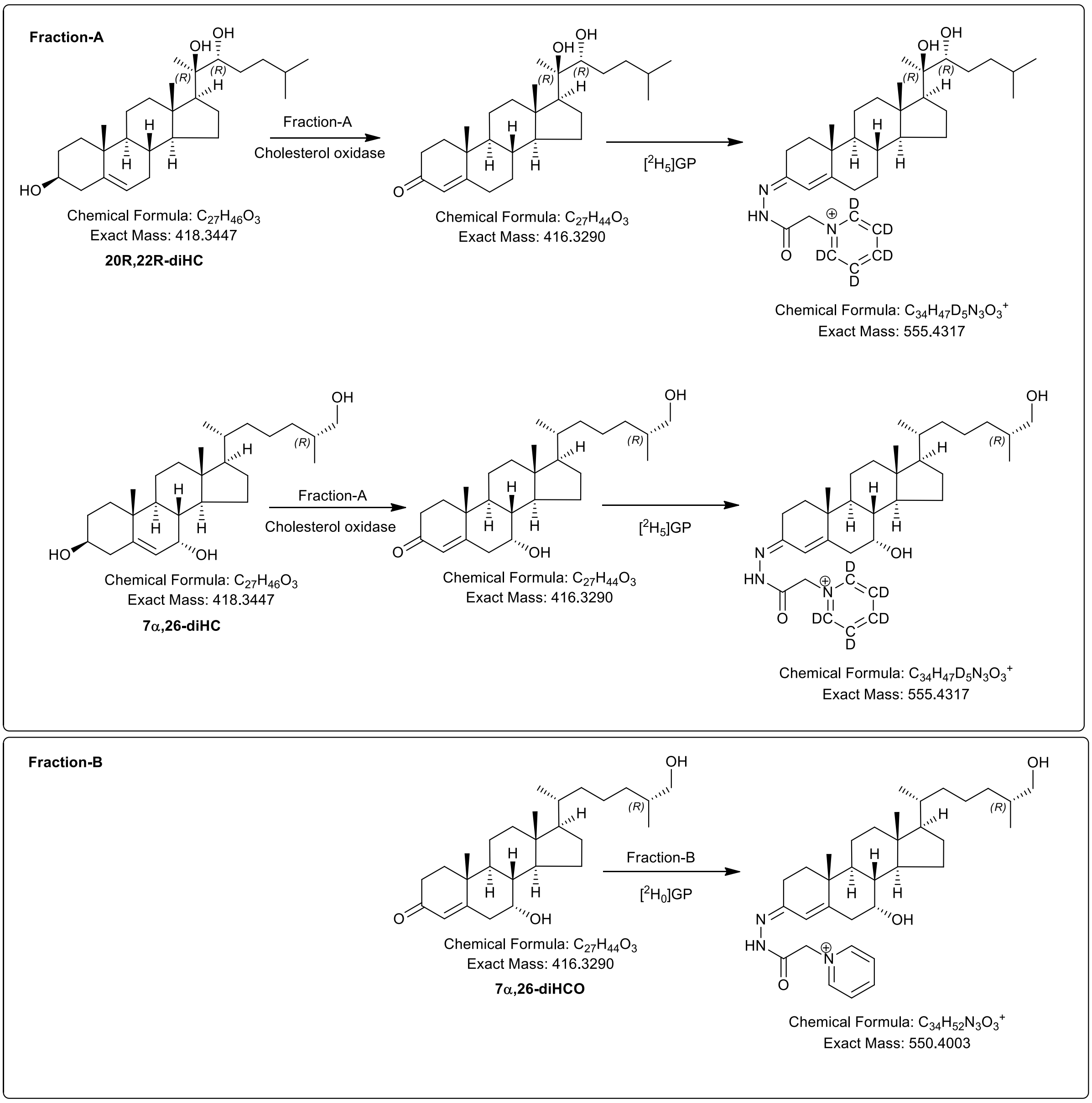
Fraction-A
20R,22R-diHC
Chemical Formula: $C_{27}H_{46}O_3$
Exact Mass: 418.3447
Fraction-A
Cholesterol oxidase
Chemical Formula: $C_{27}H_{44}O_3$
Exact Mass: 416.3290
[$^2H_5$]GP
Chemical Formula: $C_{34}H_{47}D_5N_3O_3^+$
Exact Mass: 555.4317
7α,26-diHC
Chemical Formula: $C_{27}H_{46}O_3$
Exact Mass: 418.3447
Fraction-A
Cholesterol oxidase
Chemical Formula: $C_{27}H_{44}O_3$
Exact Mass: 416.3290
[$^2H_5$]GP
Chemical Formula: $C_{34}H_{47}D_5N_3O_3^+$
Exact Mass: 555.4317
Fraction-B
7α,26-diHCO
Chemical Formula: $C_{27}H_{44}O_3$
Exact Mass: 416.3290
Fraction-B
[$^2H_0$]GP
Chemical Formula: $C_{34}H_{52}N_3O_3^+$
Exact Mass: 550.4003

Figure S1B

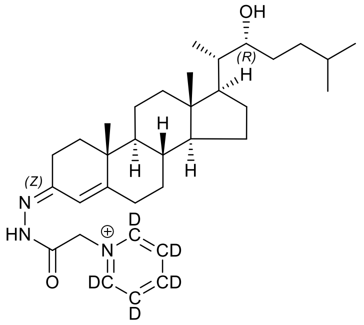

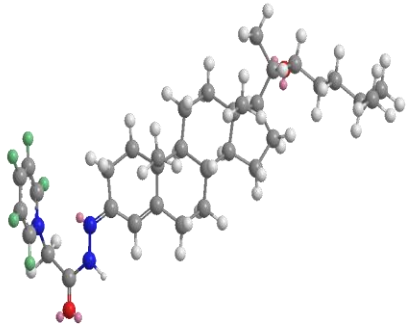

Chemical Formula: $C_{34}H_{47}D_5N_3O_2^+$
Exact Mass: 539.4368

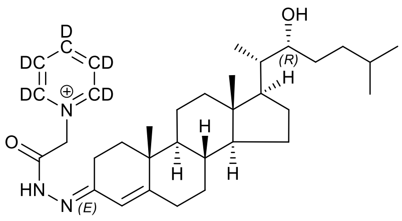

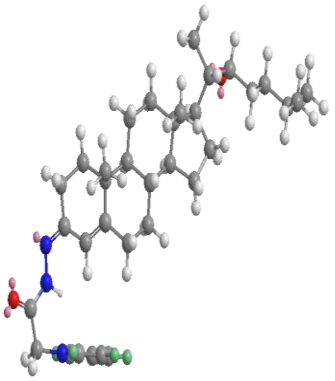

Chemical Formula: $C_{34}H_{47}D_5N_3O_2^+$
Exact Mass: 539.4368

Figure S1C

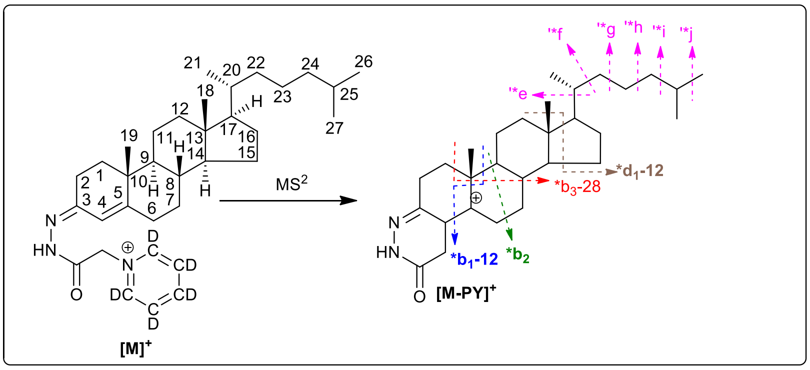
MS2
[M]+
[M-PY]+
*b1-12
*b2
*b3-28
*d1-12
**e
**f
**g
**h
**i
**j

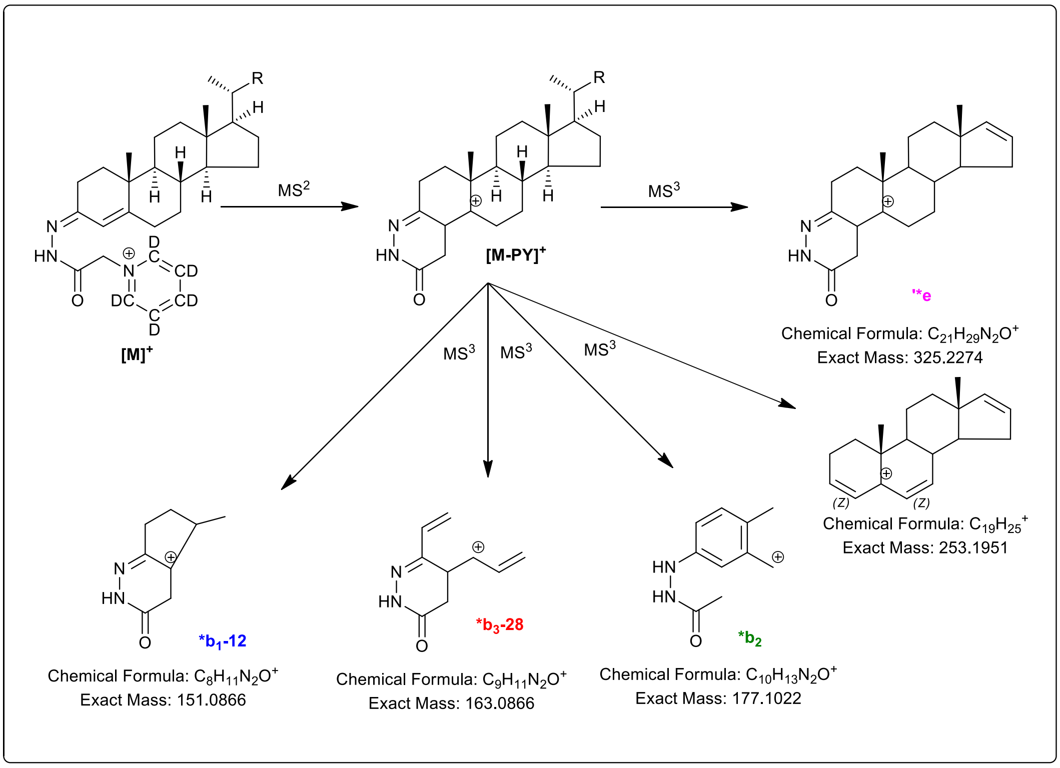
MS2
MS3
[M]+
[M-PY]+
**e
Chemical Formula: C21H29N2O+
Exact Mass: 325.2274
Chemical Formula: C19H25+
Exact Mass: 253.1951
*b1-12
Chemical Formula: C8H11N2O+
Exact Mass: 151.0866
*b3-28
Chemical Formula: C9H11N2O+
Exact Mass: 163.0866
*b2
Chemical Formula: C10H13N2O+
Exact Mass: 177.1022

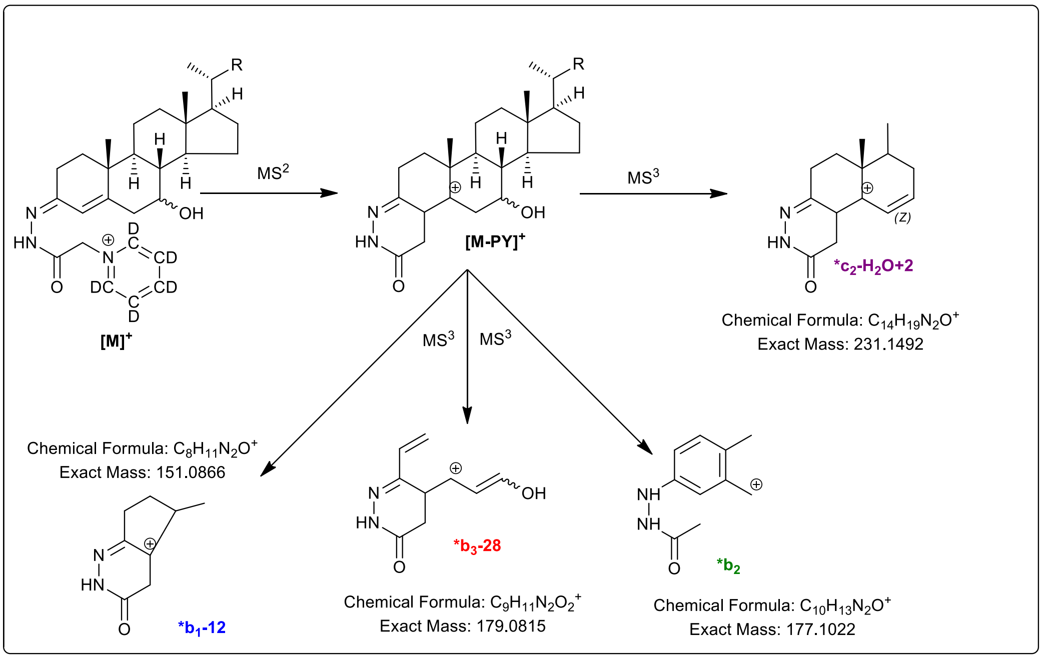
MS2
MS3
[M]+
[M-PY]+
*c2-H2O+2
Chemical Formula: C14H19N2O+
Exact Mass: 231.1492
Chemical Formula: C8H11N2O+
Exact Mass: 151.0866
*b1-12
*b3-28
Chemical Formula: C9H11N2O2+
Exact Mass: 179.0815
*b2
Chemical Formula: C10H13N2O+
Exact Mass: 177.1022

Figure S1D

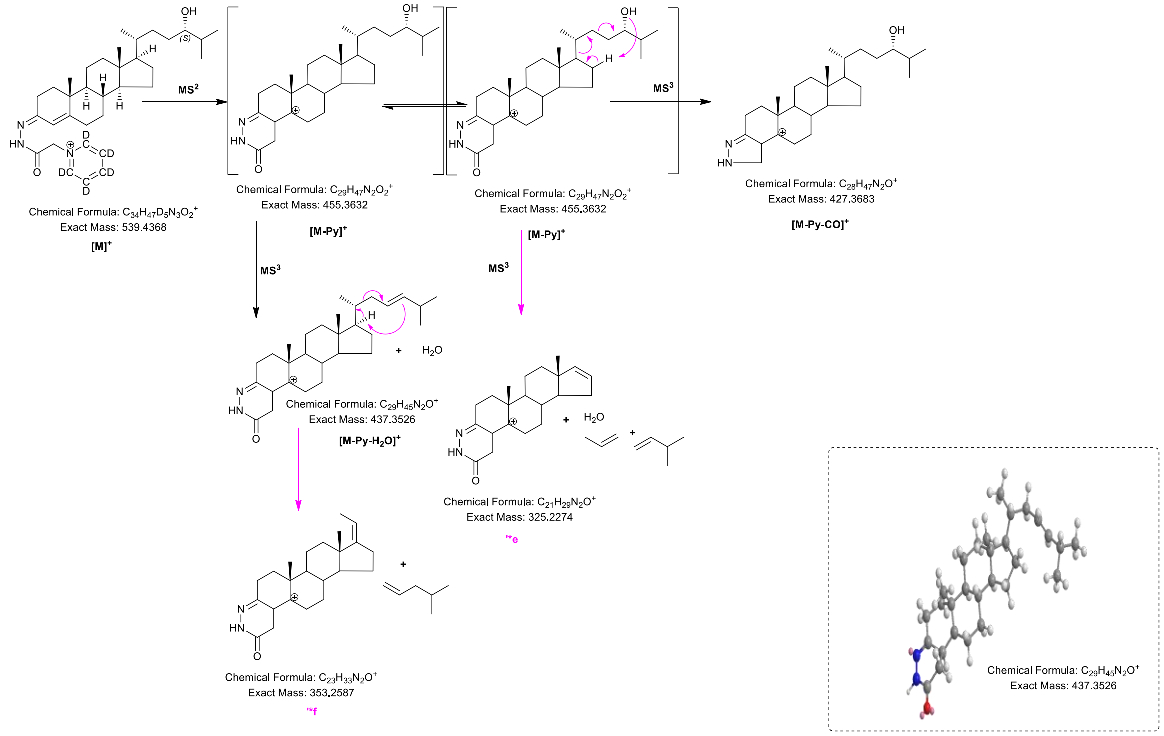

OH
(S)
H
MS2
MS3
N
HN
O
Chemical Formula: C34H47D5N3O2+
Exact Mass: 539.4368
[M]+
Chemical Formula: C29H47N2O2+
Exact Mass: 455.3632
[M-Py]+
Chemical Formula: C28H47N2O+
Exact Mass: 427.3683
[M-Py-CO]+
+ H2O
Chemical Formula: C29H45N2O+
Exact Mass: 437.3526
[M-Py-H2O]+
Chemical Formula: C21H29N2O+
Exact Mass: 325.2274
**e
Chemical Formula: C23H33N2O+
Exact Mass: 353.2587
**f
Chemical Formula: C29H45N2O+
Exact Mass: 437.3526

Figure S2A

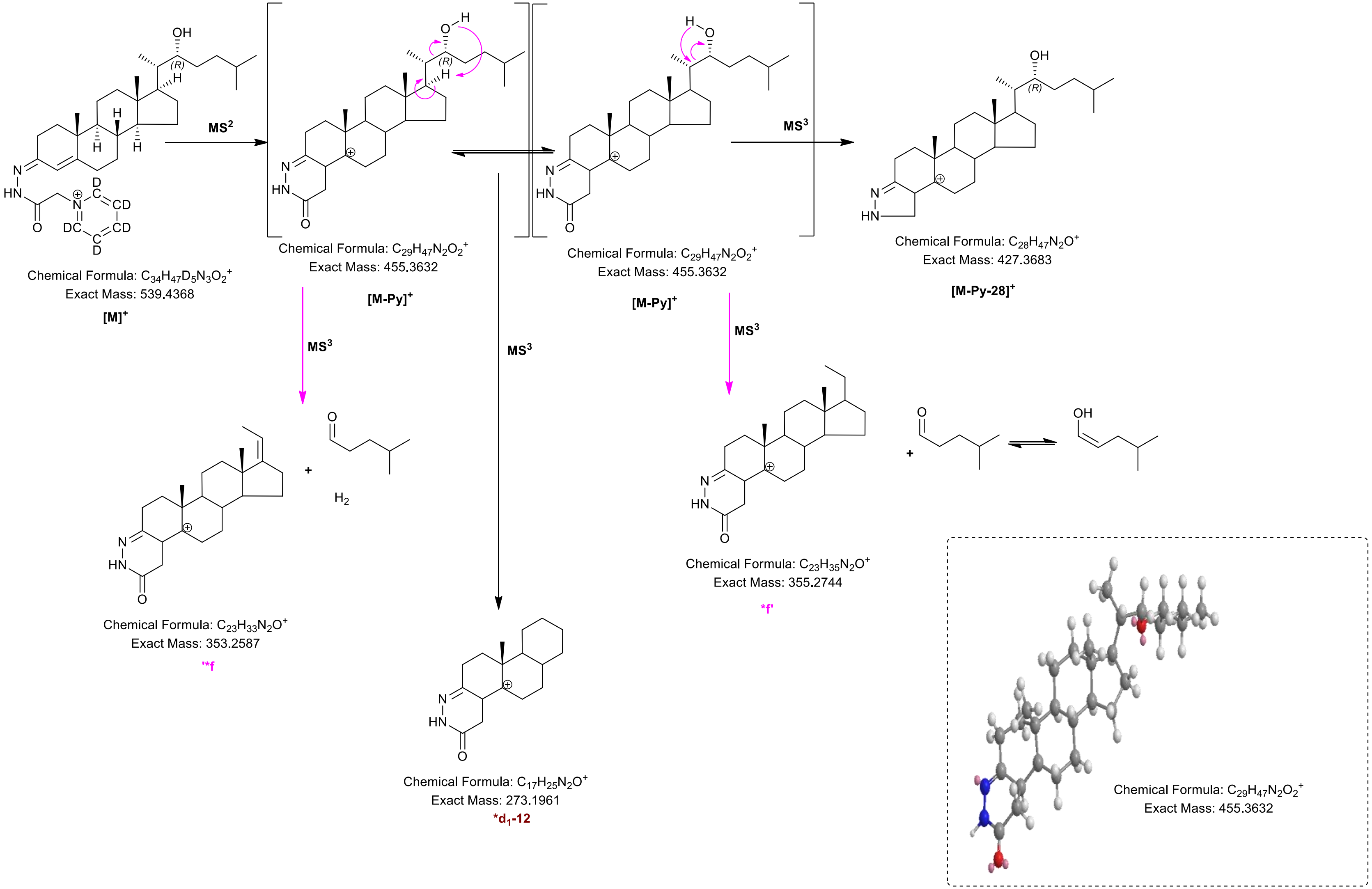

OH
(R)
H
MS2
Chemical Formula: C34H47D5N3O2+
Exact Mass: 539.4368
[M]+
Chemical Formula: C29H47N2O2+
Exact Mass: 455.3632
[M-Py]+
Chemical Formula: C29H47N2O2+
Exact Mass: 455.3632
[M-Py]+
MS3
Chemical Formula: C28H47N2O+
Exact Mass: 427.3683
[M-Py-28]+
MS3
MS3
MS3
H2
Chemical Formula: C23H33N2O+
Exact Mass: 353.2587
**f
Chemical Formula: C17H25N2O+
Exact Mass: 273.1961
*d1-12
Chemical Formula: C23H35N2O+
Exact Mass: 355.2744
*f'
Chemical Formula: C29H47N2O2+
Exact Mass: 455.3632

Figure S2B

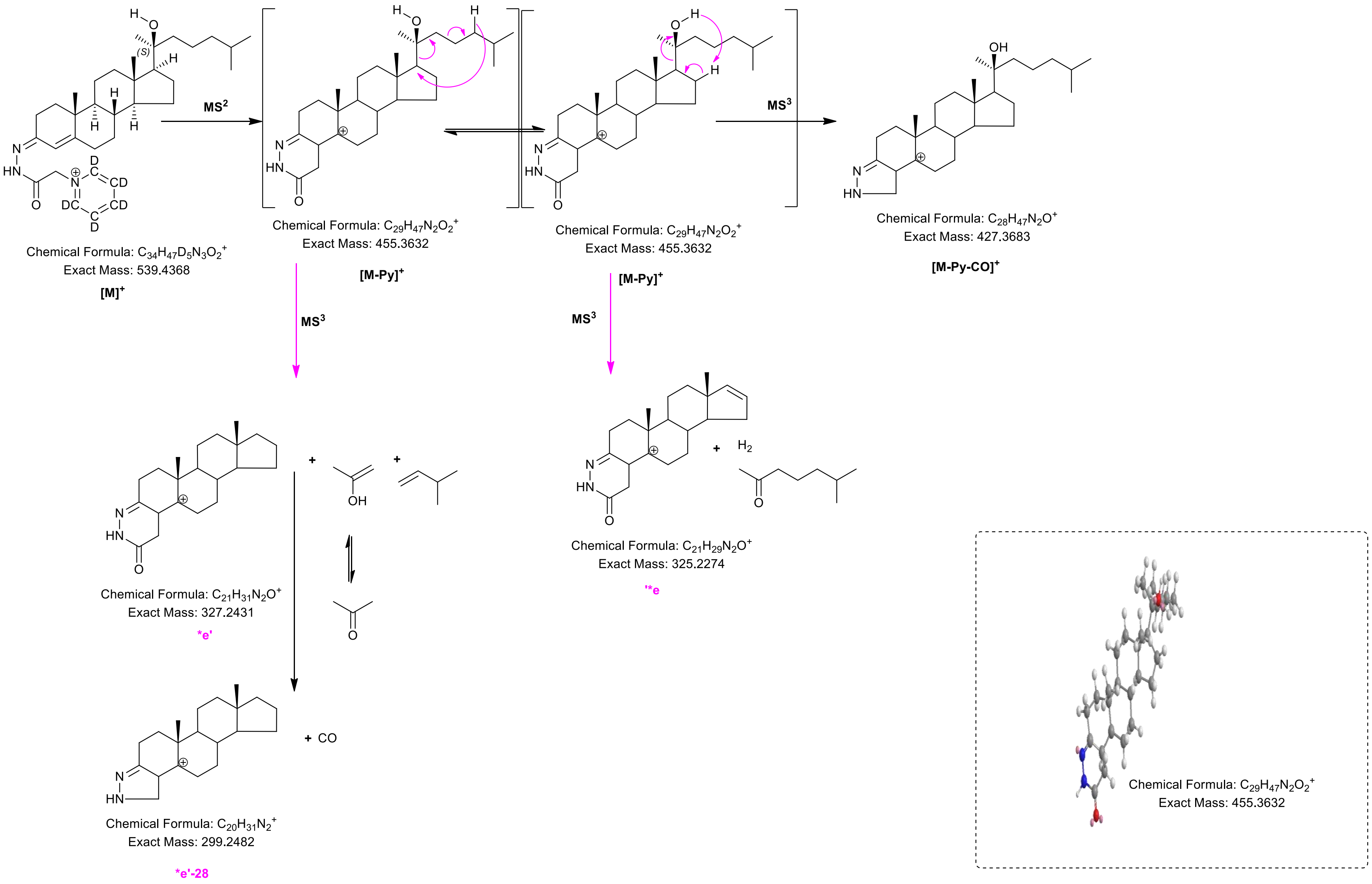
MS^2
MS^3
Chemical Formula: $C_{34}H_{47}D_5N_3O_2^+$
Exact Mass: 539.4368
[M]$^+$
Chemical Formula: $C_{29}H_{47}N_2O_2^+$
Exact Mass: 455.3632
[M-Py]$^+$
Chemical Formula: $C_{29}H_{47}N_2O_2^+$
Exact Mass: 455.3632
[M-Py]$^+$
Chemical Formula: $C_{28}H_{47}N_2O^+$
Exact Mass: 427.3683
[M-Py-CO]$^+$
MS^3
MS^3
Chemical Formula: $C_{21}H_{31}N_2O^+$
Exact Mass: 327.2431
*e'
Chemical Formula: $C_{21}H_{29}N_2O^+$
Exact Mass: 325.2274
'*e
+ CO
Chemical Formula: $C_{20}H_{31}N_2^+$
Exact Mass: 299.2482
*e'-28
Chemical Formula: $C_{29}H_{47}N_2O_2^+$
Exact Mass: 455.3632

Figure S2B

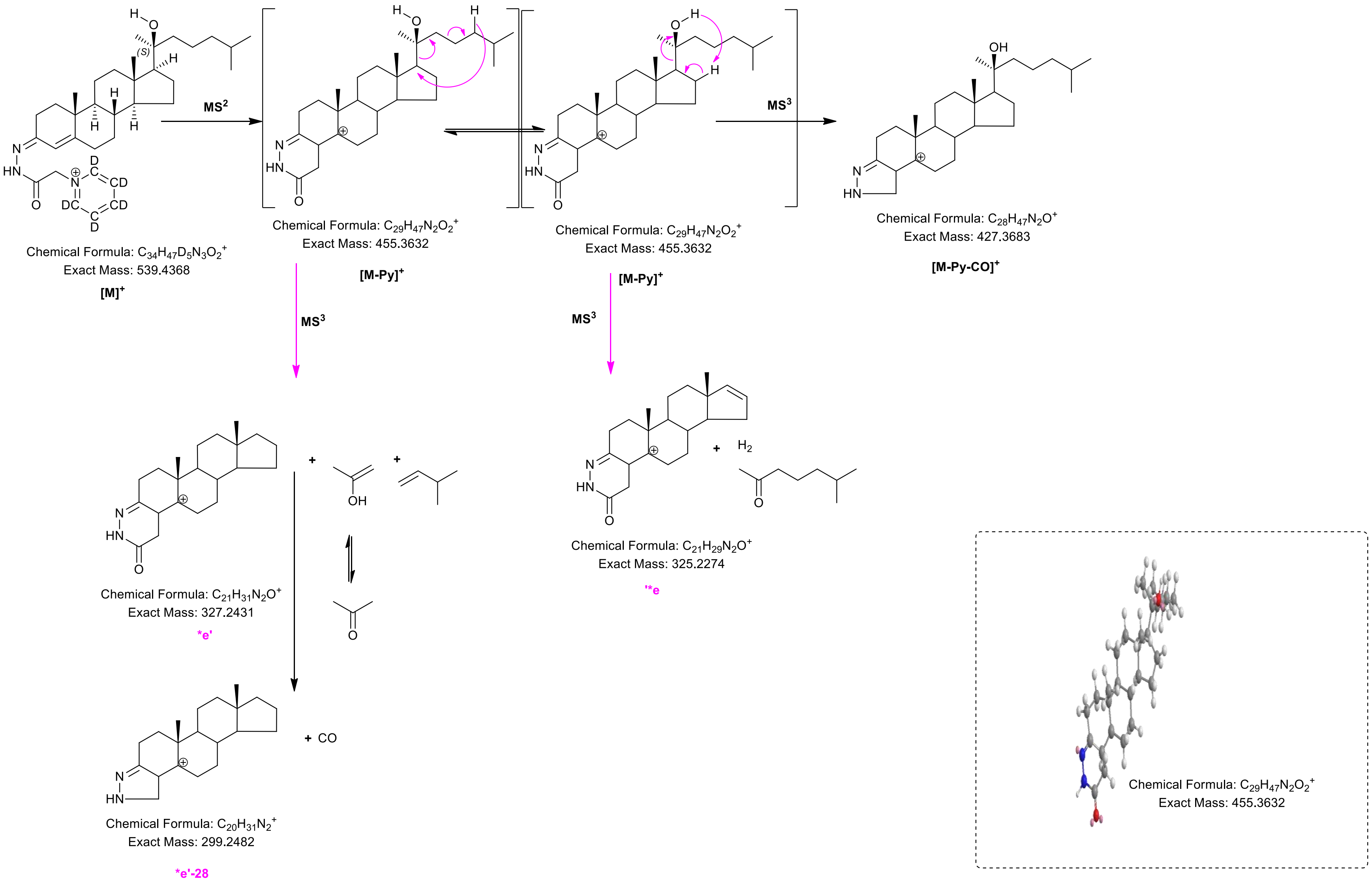

MS2
Chemical Formula: C34H47D5N3O2+
Exact Mass: 539.4368
[M]+
Chemical Formula: C29H47N2O2+
Exact Mass: 455.3632
[M-Py]+
Chemical Formula: C29H47N2O2+
Exact Mass: 455.3632
[M-Py]+
MS3
Chemical Formula: C28H47N2O+
Exact Mass: 427.3683
[M-Py-CO]+
MS3
MS3
Chemical Formula: C21H31N2O+
Exact Mass: 327.2431
*e'
Chemical Formula: C21H29N2O+
Exact Mass: 325.2274
'*e
+ CO
Chemical Formula: C20H31N2+
Exact Mass: 299.2482
*e'-28
Chemical Formula: C29H47N2O2+
Exact Mass: 455.3632

Figure S2C

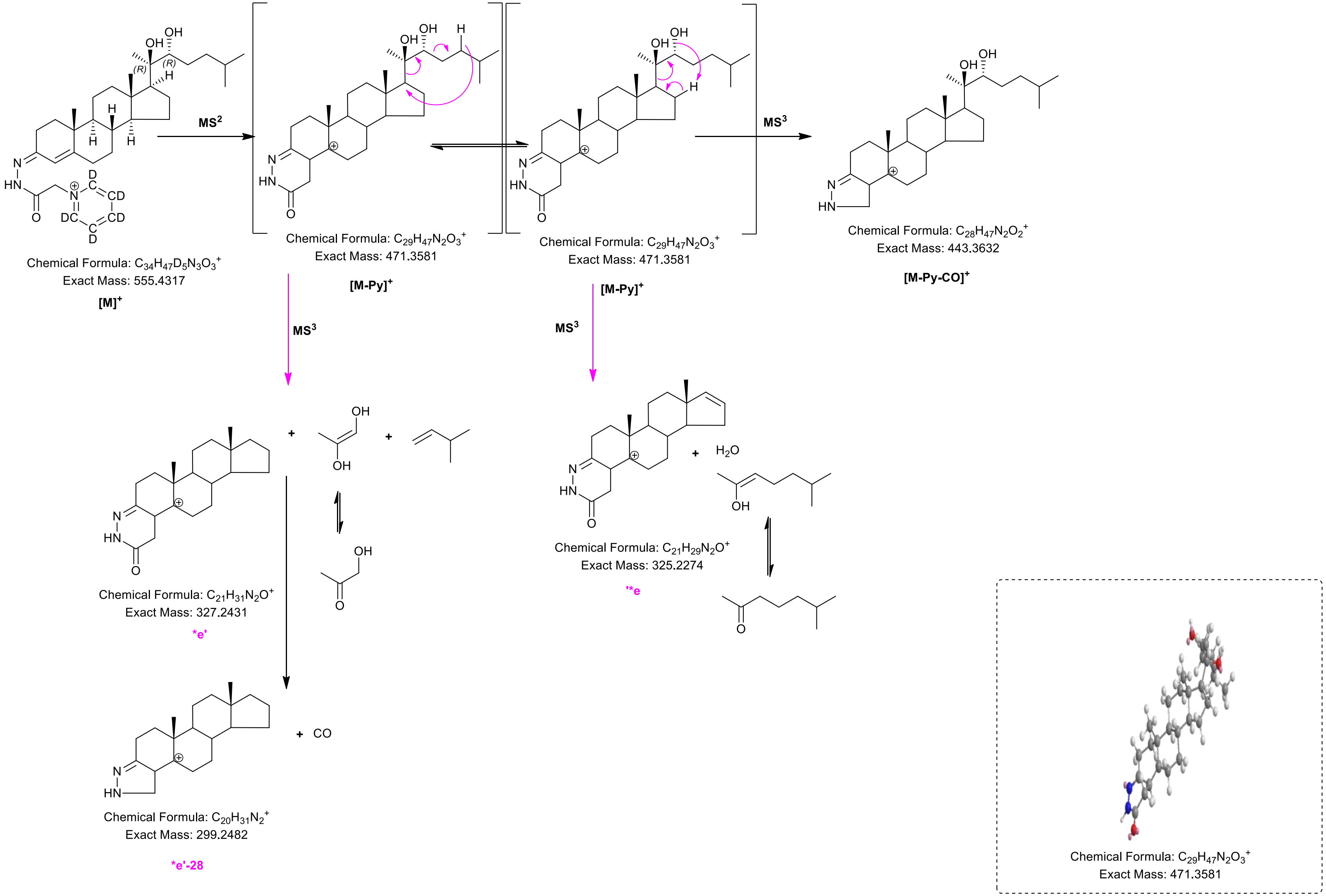
MS2
MS3
MS3
MS3
Chemical Formula: C34H47D5N3O3+
Exact Mass: 555.4317
[M]+
Chemical Formula: C29H47N2O3+
Exact Mass: 471.3581
[M-Py]+
Chemical Formula: C29H47N2O3+
Exact Mass: 471.3581
[M-Py]+
Chemical Formula: C28H47N2O2+
Exact Mass: 443.3632
[M-Py-CO]+
Chemical Formula: C21H31N2O+
Exact Mass: 327.2431
*e'
Chemical Formula: C21H29N2O+
Exact Mass: 325.2274
**e
Chemical Formula: C20H31N2+
Exact Mass: 299.2482
*e'-28
+ CO
H2O
Chemical Formula: C29H47N2O3+
Exact Mass: 471.3581

Figure S2D

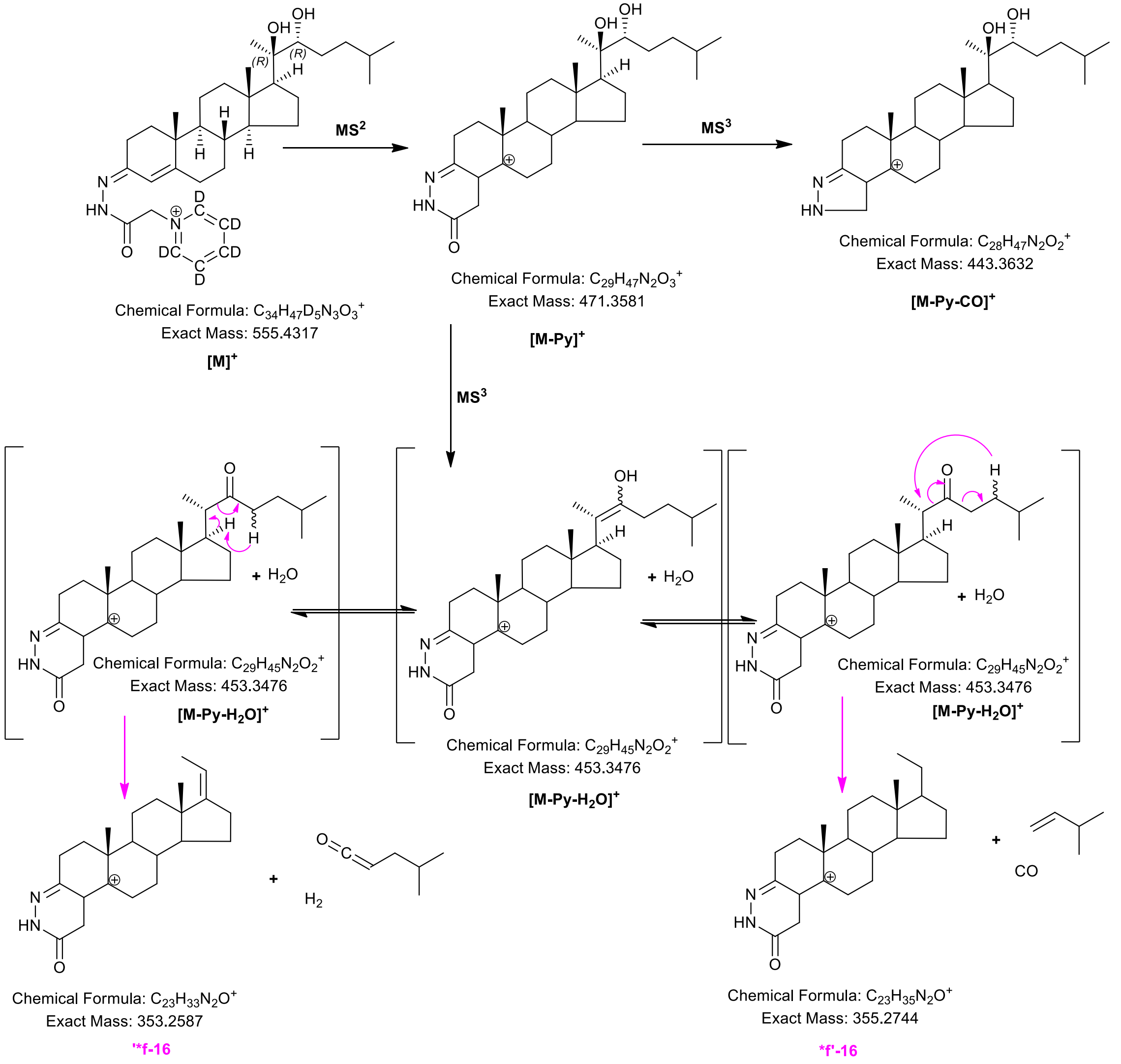
MS2
MS3
MS3
Chemical Formula: C34H47D5N3O3+
Exact Mass: 555.4317
[M]+
Chemical Formula: C29H47N2O3+
Exact Mass: 471.3581
[M-Py]+
Chemical Formula: C28H47N2O2+
Exact Mass: 443.3632
[M-Py-CO]+
+ H2O
Chemical Formula: C29H45N2O2+
Exact Mass: 453.3476
[M-Py-H2O]+
+ H2O
Chemical Formula: C29H45N2O2+
Exact Mass: 453.3476
[M-Py-H2O]+
+ H2O
Chemical Formula: C29H45N2O2+
Exact Mass: 453.3476
[M-Py-H2O]+
+
H2
Chemical Formula: C23H33N2O+
Exact Mass: 353.2587
'*f-16
+
CO
Chemical Formula: C23H35N2O+
Exact Mass: 355.2744
*f'-16

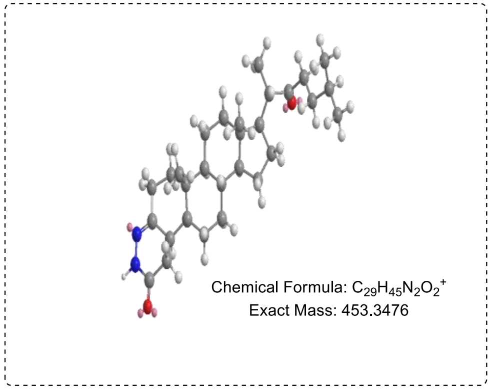
Chemical Formula: C29H45N2O2+
Exact Mass: 453.3476

Figure S2E

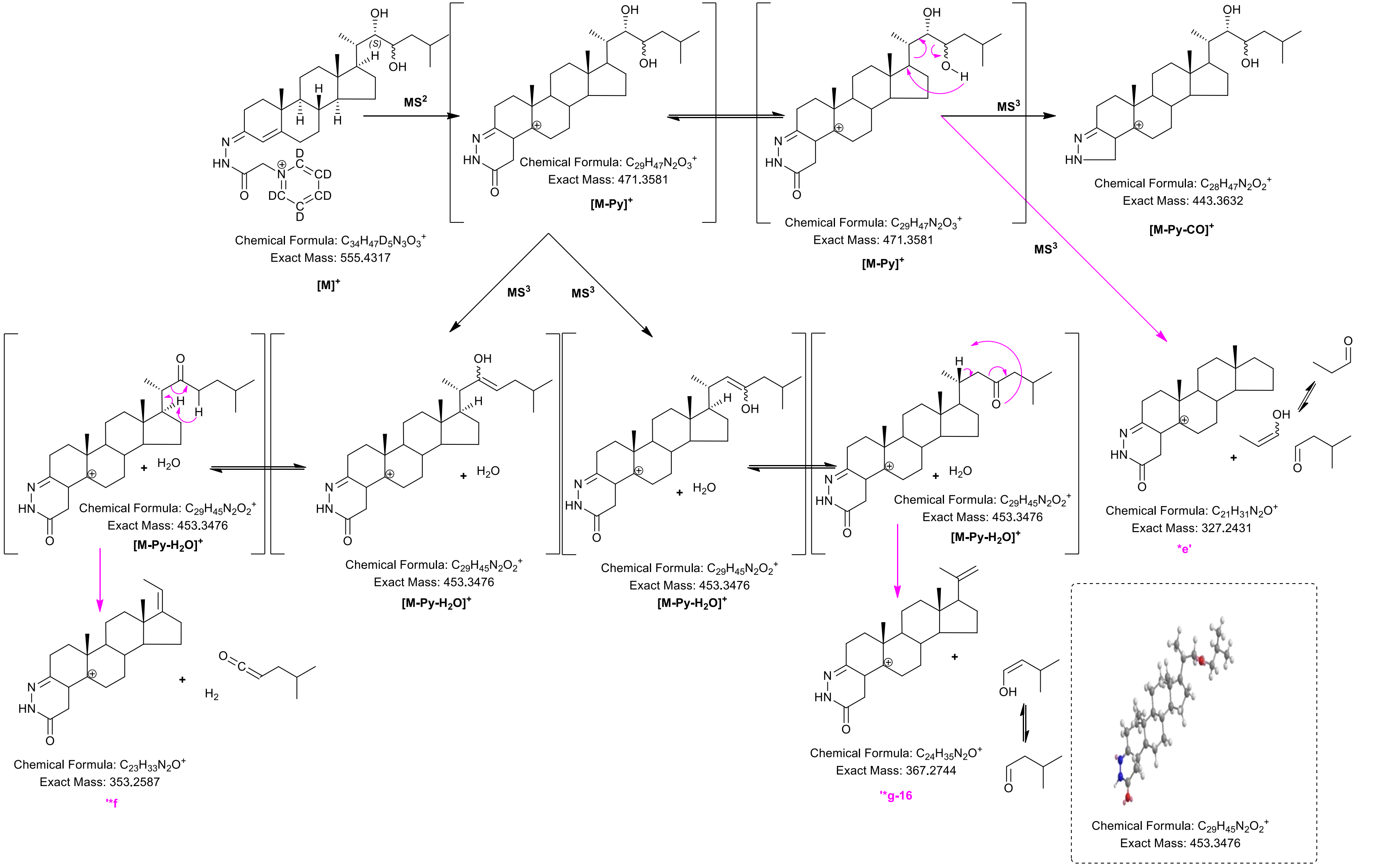
MS^2
MS^3
Chemical Formula: $C_{34}H_{47}D_5N_3O_3^+$
Exact Mass: 555.4317
[M]$^+$
Chemical Formula: $C_{29}H_{47}N_2O_3^+$
Exact Mass: 471.3581
[M-Py]$^+$
Chemical Formula: $C_{29}H_{47}N_2O_3^+$
Exact Mass: 471.3581
[M-Py]$^+$
Chemical Formula: $C_{28}H_{47}N_2O_2^+$
Exact Mass: 443.3632
[M-Py-CO]$^+$
Chemical Formula: $C_{29}H_{45}N_2O_2^+$
Exact Mass: 453.3476
[M-Py-H$_2$O]$^+$
Chemical Formula: $C_{29}H_{45}N_2O_2^+$
Exact Mass: 453.3476
[M-Py-H$_2$O]$^+$
Chemical Formula: $C_{29}H_{45}N_2O_2^+$
Exact Mass: 453.3476
[M-Py-H$_2$O]$^+$
Chemical Formula: $C_{29}H_{45}N_2O_2^+$
Exact Mass: 453.3476
[M-Py-H$_2$O]$^+$
Chemical Formula: $C_{21}H_{31}N_2O^+$
Exact Mass: 327.2431
*e'
Chemical Formula: $C_{23}H_{33}N_2O^+$
Exact Mass: 353.2587
'*f
Chemical Formula: $C_{24}H_{35}N_2O^+$
Exact Mass: 367.2744
'*g-16
Chemical Formula: $C_{29}H_{45}N_2O_2^+$
Exact Mass: 453.3476

Figure S2F

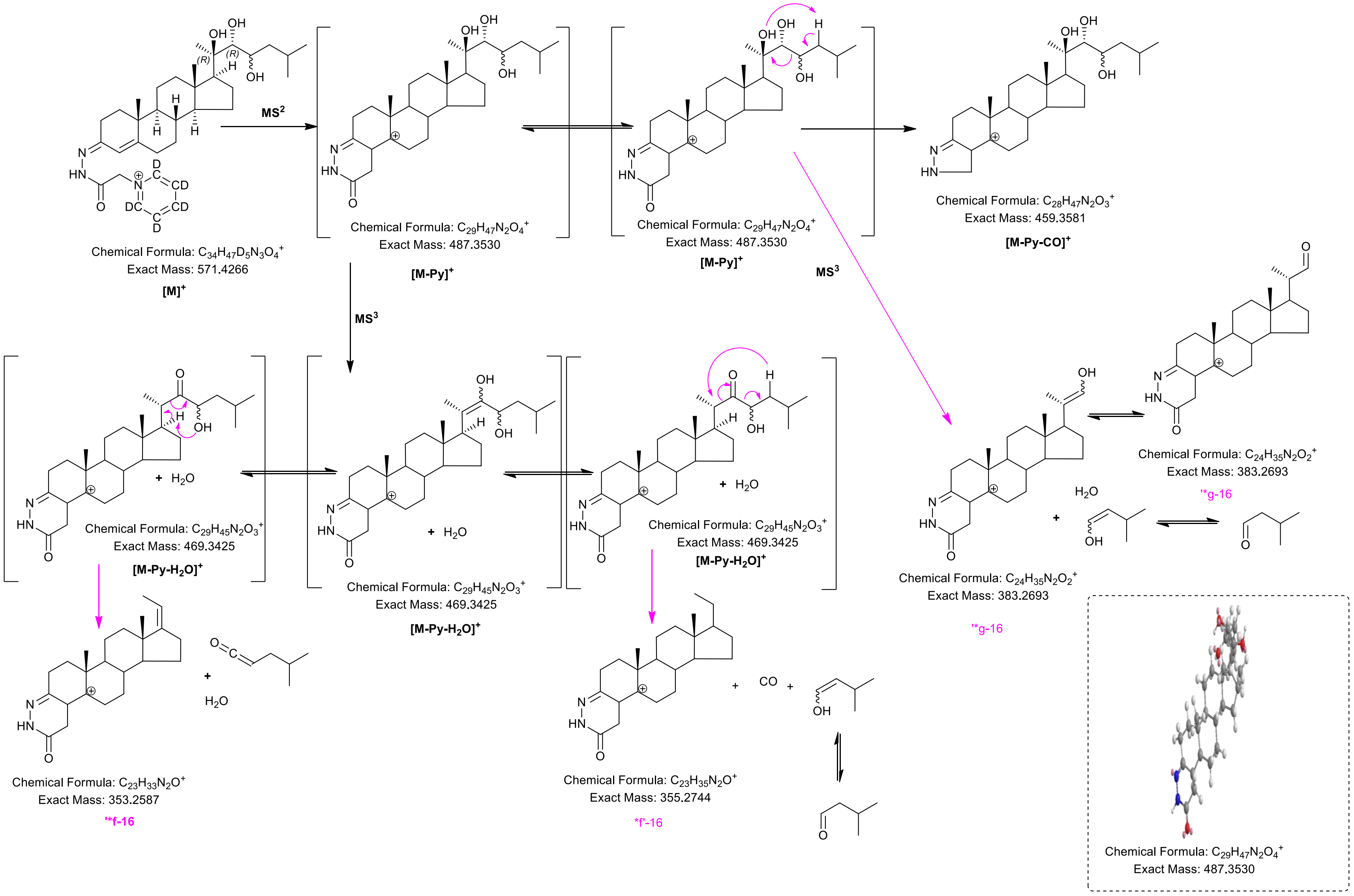
MS2
MS3
MS3
Chemical Formula: C34H47D5N3O4+
Exact Mass: 571.4266
[M]+
Chemical Formula: C29H47N2O4+
Exact Mass: 487.3530
[M-Py]+
Chemical Formula: C29H47N2O4+
Exact Mass: 487.3530
[M-Py]+
Chemical Formula: C28H47N2O3+
Exact Mass: 459.3581
[M-Py-CO]+
Chemical Formula: C24H35N2O2+
Exact Mass: 383.2693
'*g-16
Chemical Formula: C29H45N2O3+
Exact Mass: 469.3425
[M-Py-H2O]+
Chemical Formula: C29H45N2O3+
Exact Mass: 469.3425
[M-Py-H2O]+
Chemical Formula: C29H45N2O3+
Exact Mass: 469.3425
[M-Py-H2O]+
Chemical Formula: C24H35N2O2+
Exact Mass: 383.2693
'*g-16
Chemical Formula: C23H33N2O+
Exact Mass: 353.2587
'*f-16
Chemical Formula: C23H35N2O+
Exact Mass: 355.2744
*f'-16
Chemical Formula: C29H47N2O4+
Exact Mass: 487.3530

Figure S2G

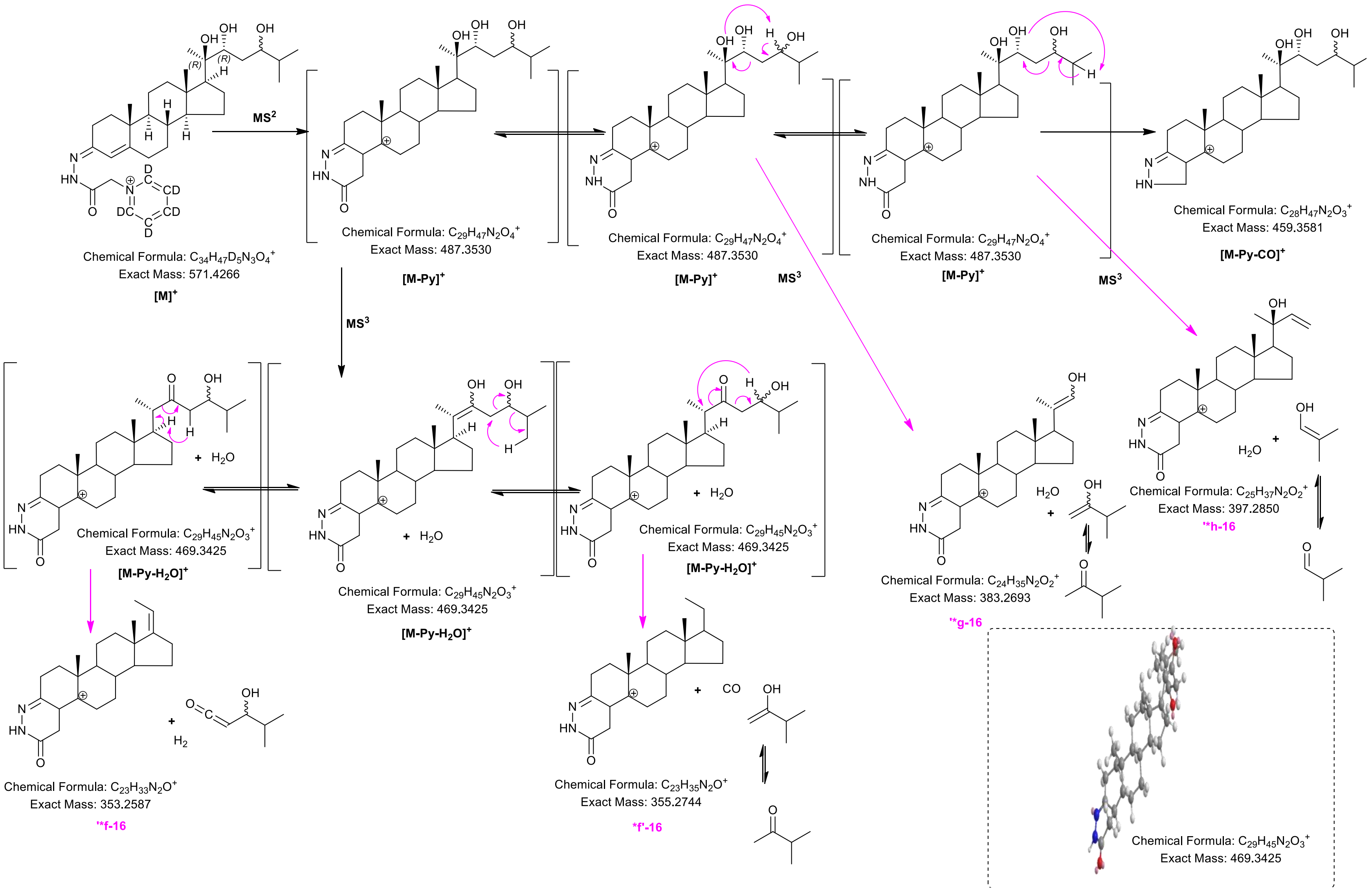

MS2
MS3
Chemical Formula: C34H47D5N3O4+
Exact Mass: 571.4266
[M]+
Chemical Formula: C29H47N2O4+
Exact Mass: 487.3530
[M-Py]+
Chemical Formula: C28H47N2O3+
Exact Mass: 459.3581
[M-Py-CO]+
Chemical Formula: C29H45N2O3+
Exact Mass: 469.3425
[M-Py-H2O]+
Chemical Formula: C24H35N2O2+
Exact Mass: 383.2693
'*g-16
Chemical Formula: C25H37N2O2+
Exact Mass: 397.2850
'*h-16
Chemical Formula: C23H33N2O+
Exact Mass: 353.2587
'*f-16
Chemical Formula: C23H35N2O+
Exact Mass: 355.2744
*f'-16
Chemical Formula: C29H45N2O3+
Exact Mass: 469.3425

Figure S2H

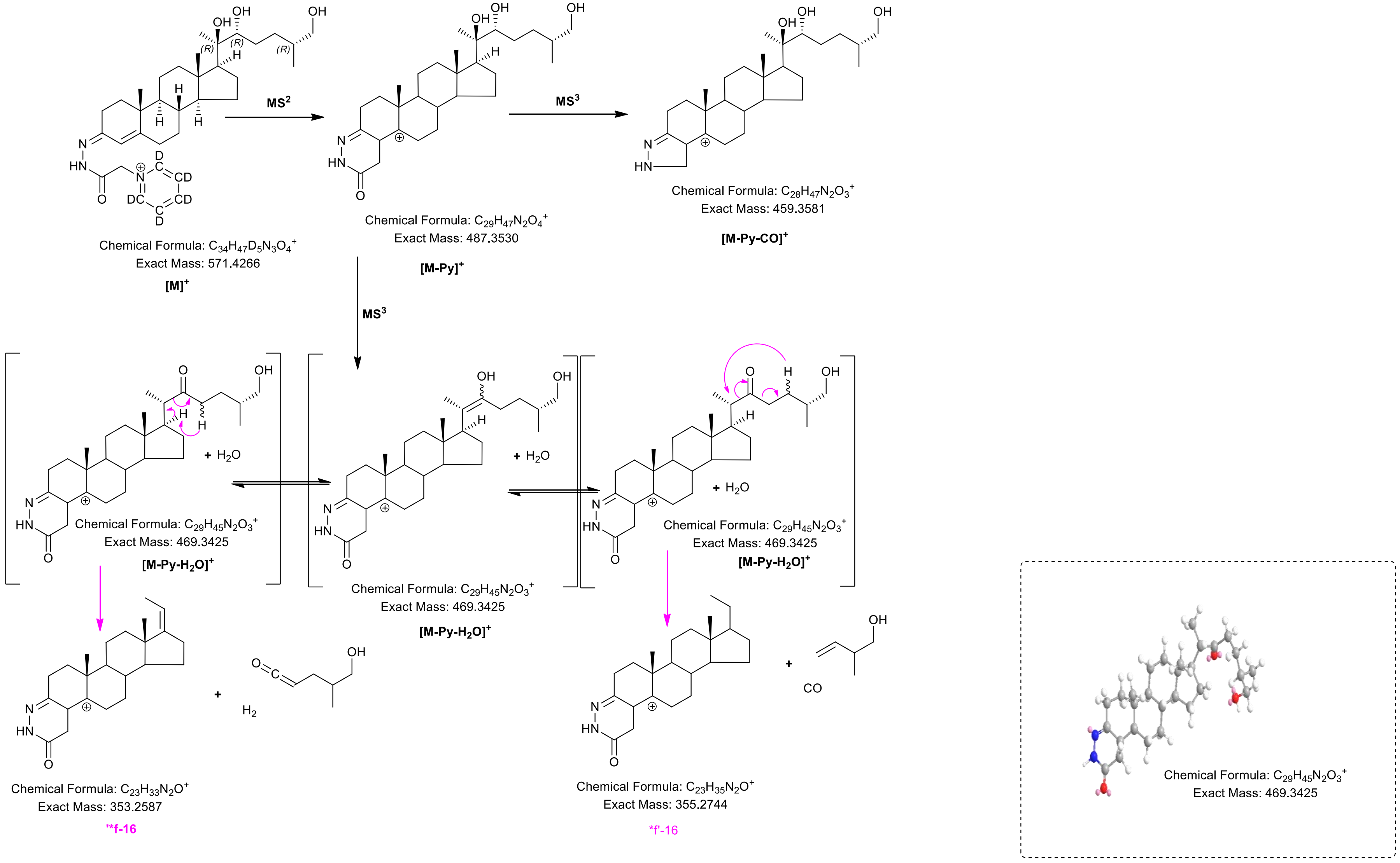
MS2
MS3
MS3
Chemical Formula: C34H47D5N3O4+
Exact Mass: 571.4266
[M]+
Chemical Formula: C29H47N2O4+
Exact Mass: 487.3530
[M-Py]+
Chemical Formula: C28H47N2O3+
Exact Mass: 459.3581
[M-Py-CO]+
+ H2O
Chemical Formula: C29H45N2O3+
Exact Mass: 469.3425
[M-Py-H2O]+
+ H2O
Chemical Formula: C29H45N2O3+
Exact Mass: 469.3425
[M-Py-H2O]+
+ H2O
Chemical Formula: C29H45N2O3+
Exact Mass: 469.3425
[M-Py-H2O]+
+ H2
Chemical Formula: C23H33N2O+
Exact Mass: 353.2587
'*f-16
+ CO
Chemical Formula: C23H35N2O+
Exact Mass: 355.2744
*f'-16
Chemical Formula: C29H45N2O3+
Exact Mass: 469.3425

Figure S2I

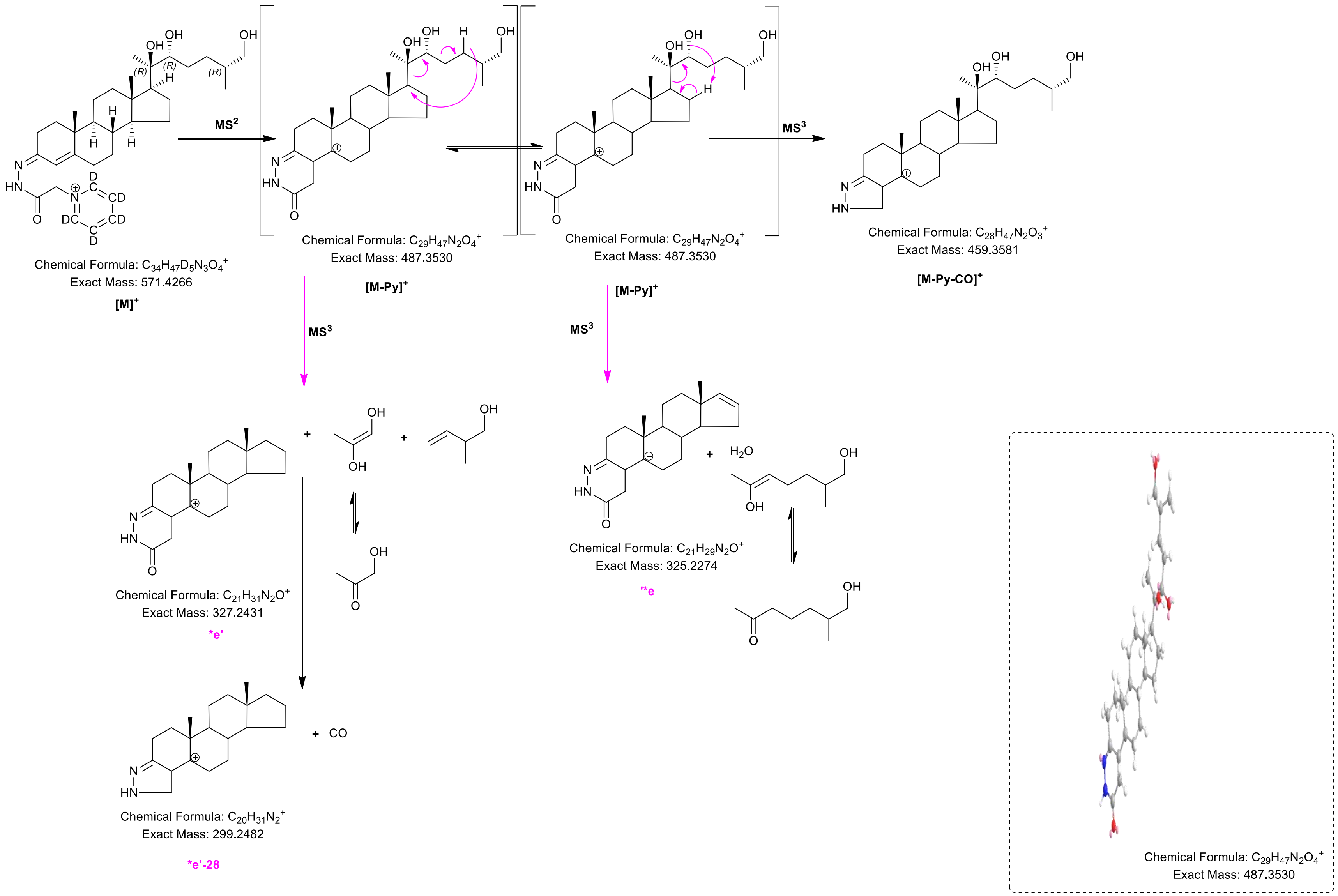
MS2
MS3
MS3
MS3
Chemical Formula: C34H47D5N3O4+
Exact Mass: 571.4266
[M]+
Chemical Formula: C29H47N2O4+
Exact Mass: 487.3530
[M-Py]+
Chemical Formula: C29H47N2O4+
Exact Mass: 487.3530
[M-Py]+
Chemical Formula: C28H47N2O3+
Exact Mass: 459.3581
[M-Py-CO]+
Chemical Formula: C21H31N2O+
Exact Mass: 327.2431
*e'
Chemical Formula: C21H29N2O+
Exact Mass: 325.2274
**e
H2O
+ CO
Chemical Formula: C20H31N2+
Exact Mass: 299.2482
*e'-28
Chemical Formula: C29H47N2O4+
Exact Mass: 487.3530

Figure S2J

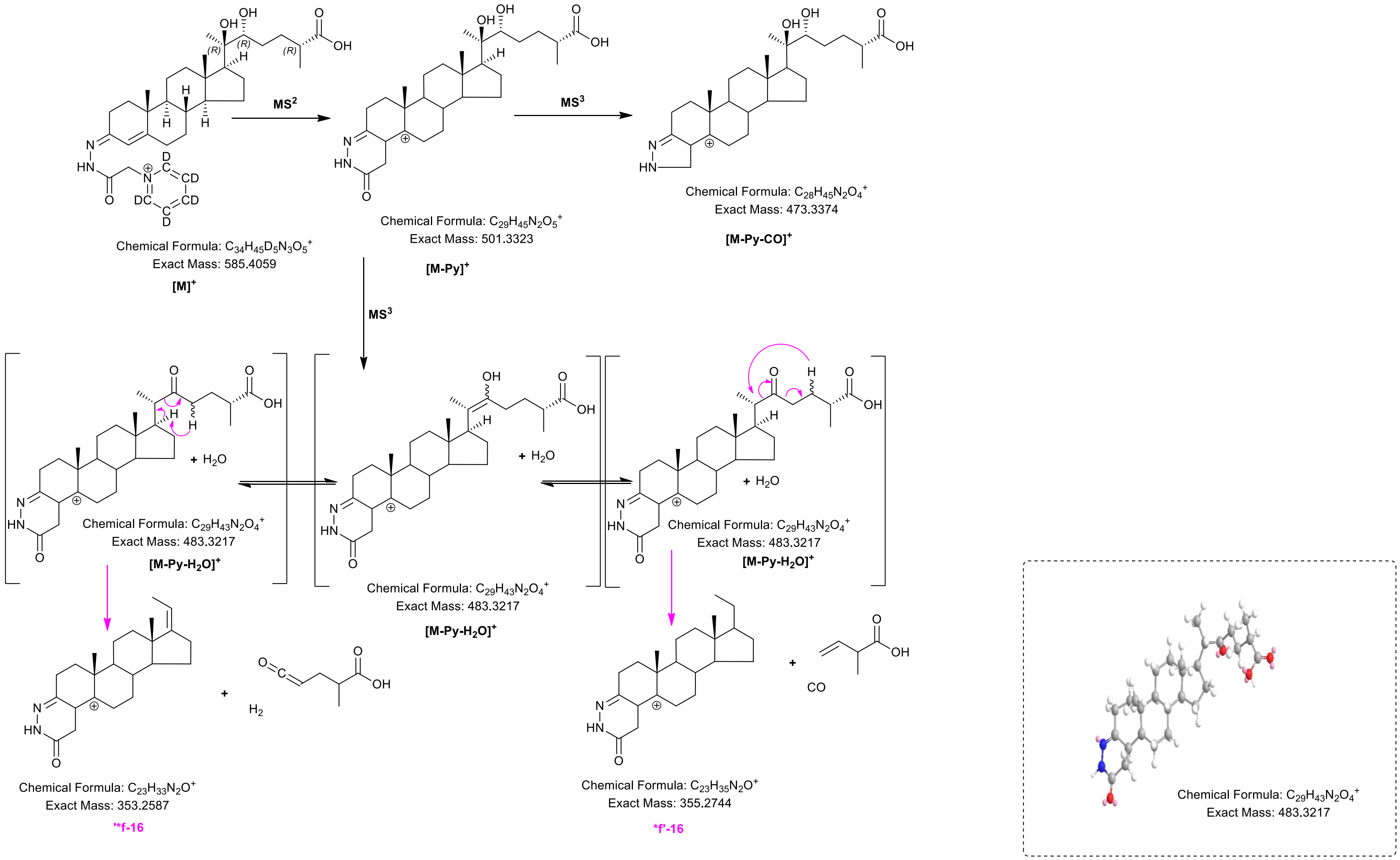
MS2
MS3
MS3
Chemical Formula: C34H45D5N3O5+
Exact Mass: 585.4059
[M]+
Chemical Formula: C29H45N2O5+
Exact Mass: 501.3323
[M-Py]+
Chemical Formula: C28H45N2O4+
Exact Mass: 473.3374
[M-Py-CO]+
+ H2O
Chemical Formula: C29H43N2O4+
Exact Mass: 483.3217
[M-Py-H2O]+
+ H2O
Chemical Formula: C29H43N2O4+
Exact Mass: 483.3217
[M-Py-H2O]+
+ H2O
Chemical Formula: C29H43N2O4+
Exact Mass: 483.3217
[M-Py-H2O]+
+ H2
Chemical Formula: C23H33N2O+
Exact Mass: 353.2587
'*f-16
+ CO
Chemical Formula: C23H35N2O+
Exact Mass: 355.2744
*f'-16
Chemical Formula: C29H43N2O4+
Exact Mass: 483.3217

Figure S2K

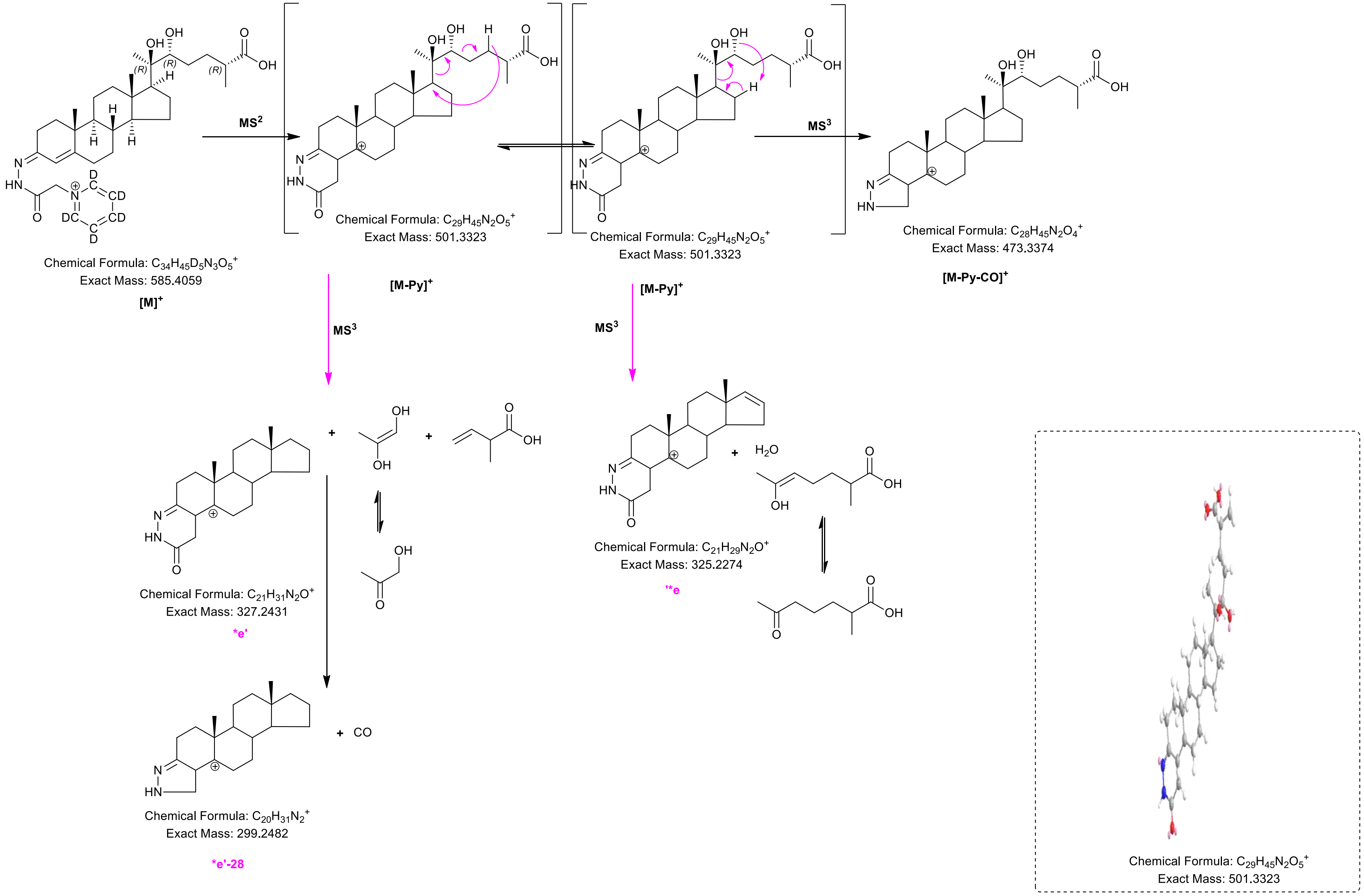
MS2
MS3
MS3
MS3
Chemical Formula: C34H45D5N3O5+
Exact Mass: 585.4059
[M]+
Chemical Formula: C29H45N2O5+
Exact Mass: 501.3323
[M-Py]+
Chemical Formula: C29H45N2O5+
Exact Mass: 501.3323
[M-Py]+
Chemical Formula: C28H45N2O4+
Exact Mass: 473.3374
[M-Py-CO]+
Chemical Formula: C21H31N2O+
Exact Mass: 327.2431
*e'
Chemical Formula: C21H29N2O+
Exact Mass: 325.2274
**e
Chemical Formula: C20H31N2+
Exact Mass: 299.2482
*e'-28
+ CO
+ H2O
Chemical Formula: C29H45N2O5+
Exact Mass: 501.3323

Figure S2L

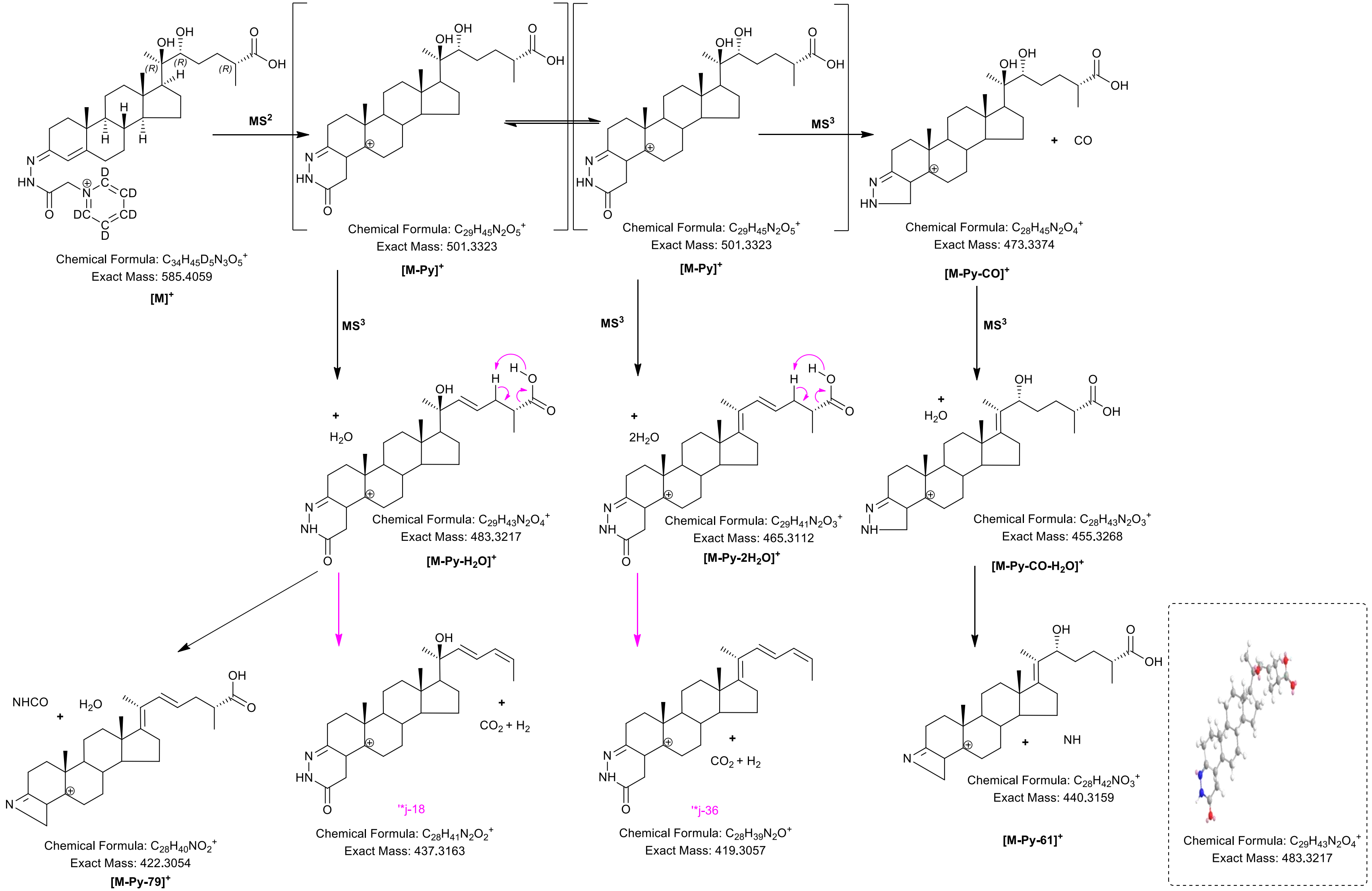

MS²
MS³
Chemical Formula: C34H45D5N3O5+
Exact Mass: 585.4059
[M]+
Chemical Formula: C29H45N2O5+
Exact Mass: 501.3323
[M-Py]+
Chemical Formula: C29H45N2O5+
Exact Mass: 501.3323
[M-Py]+
+ CO
Chemical Formula: C28H45N2O4+
Exact Mass: 473.3374
[M-Py-CO]+
+ H2O
Chemical Formula: C29H43N2O4+
Exact Mass: 483.3217
[M-Py-H2O]+
+ 2H2O
Chemical Formula: C29H41N2O3+
Exact Mass: 465.3112
[M-Py-2H2O]+
+ H2O
Chemical Formula: C28H43N2O3+
Exact Mass: 455.3268
[M-Py-CO-H2O]+
NHCO + H2O
Chemical Formula: C28H40NO2+
Exact Mass: 422.3054
[M-Py-79]+
+ CO2 + H2
'*j-18
Chemical Formula: C28H41N2O2+
Exact Mass: 437.3163
+ CO2 + H2
'*j-36
Chemical Formula: C28H39N2O+
Exact Mass: 419.3057
+ NH
Chemical Formula: C28H42NO3+
Exact Mass: 440.3159
[M-Py-61]+
Chemical Formula: C29H43N2O4+
Exact Mass: 483.3217

Figure S2M

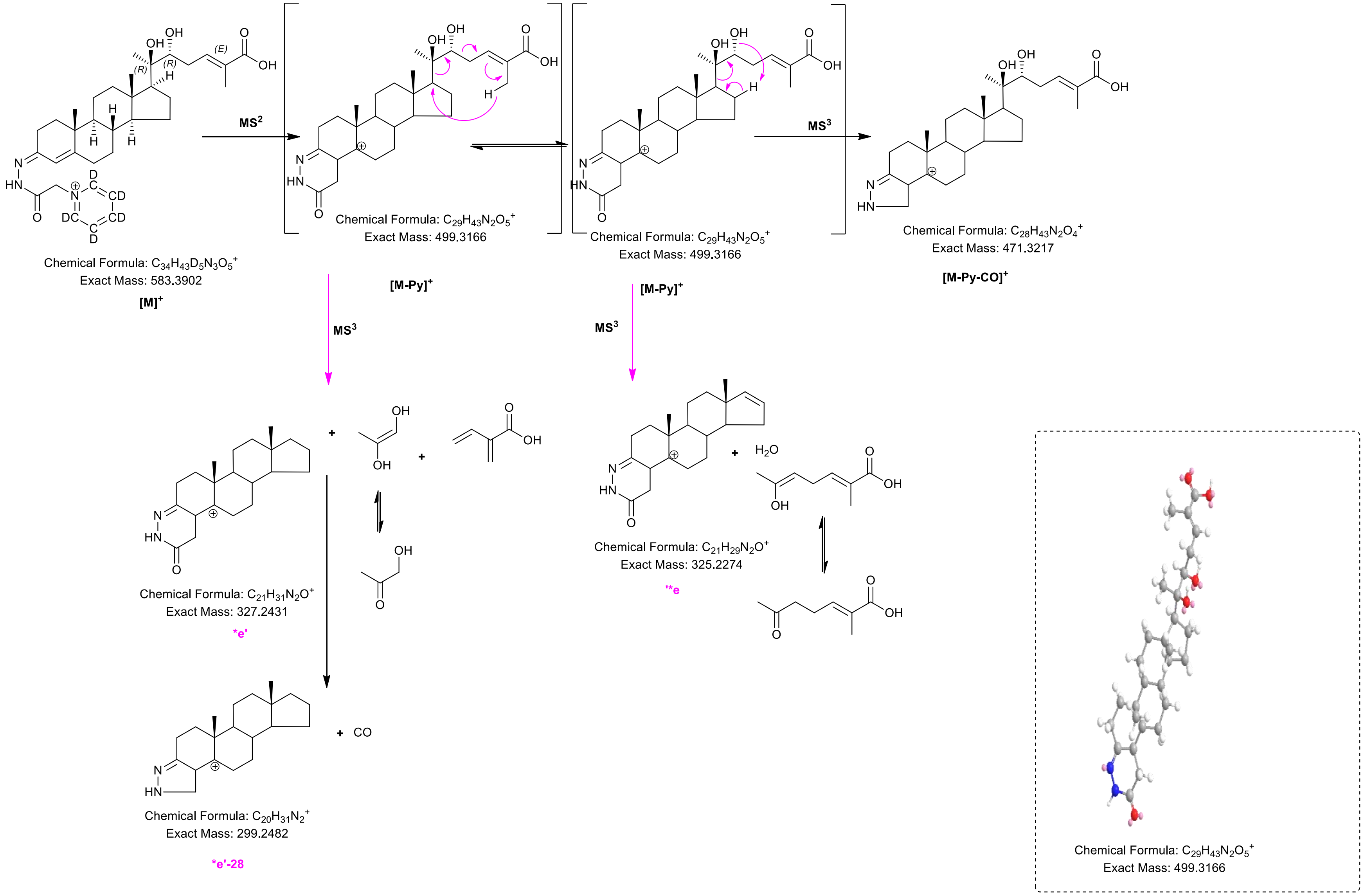
MS^2
MS^3
Chemical Formula: $C_{34}H_{43}D_5N_3O_5^+$
Exact Mass: 583.3902
[M]$^+$
Chemical Formula: $C_{29}H_{43}N_2O_5^+$
Exact Mass: 499.3166
[M-Py]$^+$
Chemical Formula: $C_{29}H_{43}N_2O_5^+$
Exact Mass: 499.3166
[M-Py]$^+$
Chemical Formula: $C_{28}H_{43}N_2O_4^+$
Exact Mass: 471.3217
[M-Py-CO]$^+$
MS^3
MS^3
Chemical Formula: $C_{21}H_{31}N_2O^+$
Exact Mass: 327.2431
*e'
Chemical Formula: $C_{20}H_{31}N_2^+$
Exact Mass: 299.2482
*e'-28
+ CO
Chemical Formula: $C_{21}H_{29}N_2O^+$
Exact Mass: 325.2274
**e
+ H_2O
Chemical Formula: $C_{29}H_{43}N_2O_5^+$
Exact Mass: 499.3166

Figure S2N

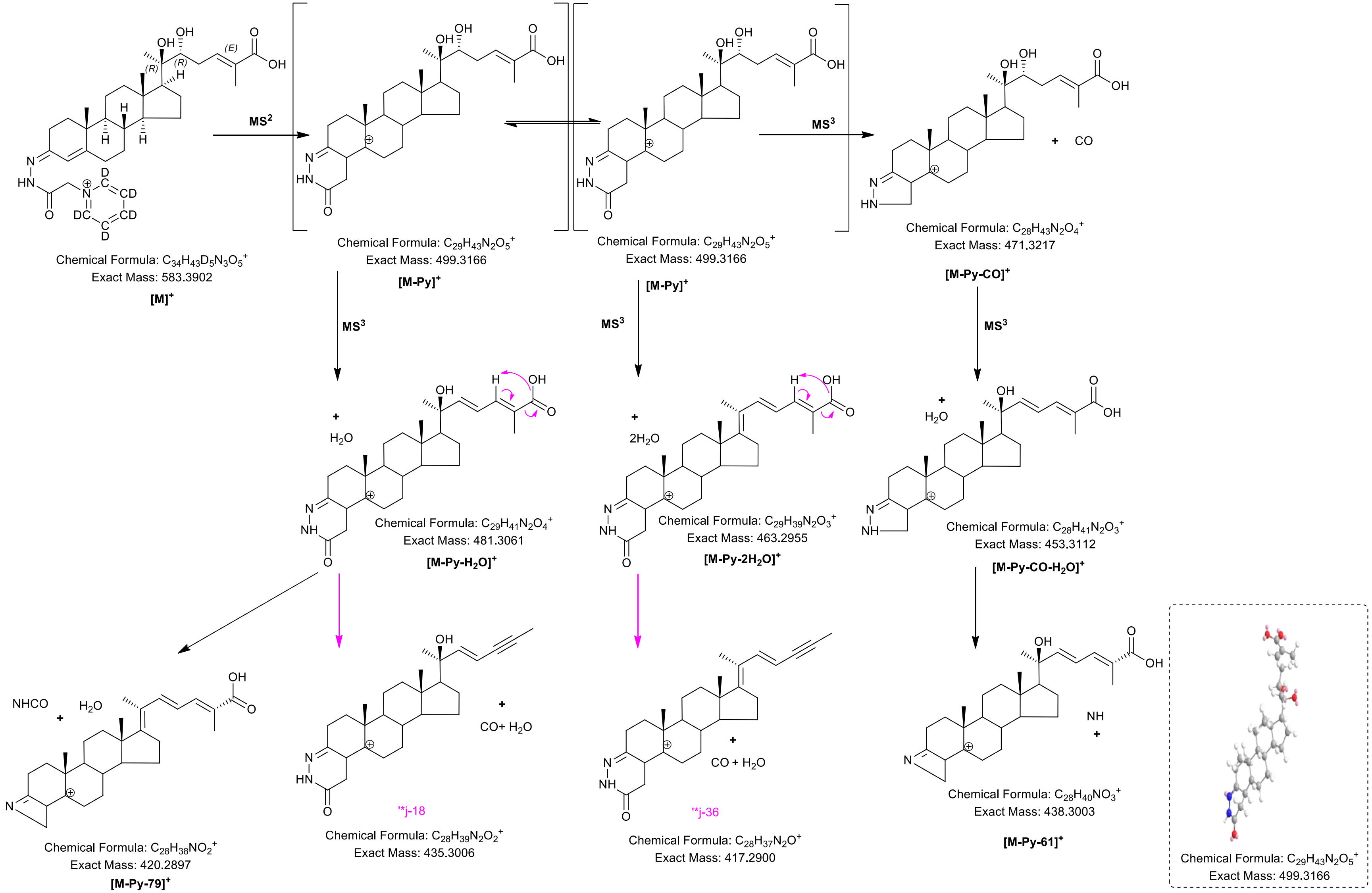

Chemical Formula: C34H43D5N3O5+
Exact Mass: 583.3902
[M]+
MS2
Chemical Formula: C29H43N2O5+
Exact Mass: 499.3166
[M-Py]+
MS3
Chemical Formula: C28H43N2O4+
Exact Mass: 471.3217
[M-Py-CO]+
Chemical Formula: C29H41N2O4+
Exact Mass: 481.3061
[M-Py-H2O]+
Chemical Formula: C29H39N2O3+
Exact Mass: 463.2955
[M-Py-2H2O]+
Chemical Formula: C28H41N2O3+
Exact Mass: 453.3112
[M-Py-CO-H2O]+
Chemical Formula: C28H38NO2+
Exact Mass: 420.2897
[M-Py-79]+
'*j-18
Chemical Formula: C28H39N2O2+
Exact Mass: 435.3006
'*j-36
Chemical Formula: C28H37N2O+
Exact Mass: 417.2900
Chemical Formula: C28H40NO3+
Exact Mass: 438.3003
[M-Py-61]+
Chemical Formula: C29H43N2O5+
Exact Mass: 499.3166

Figure S2O

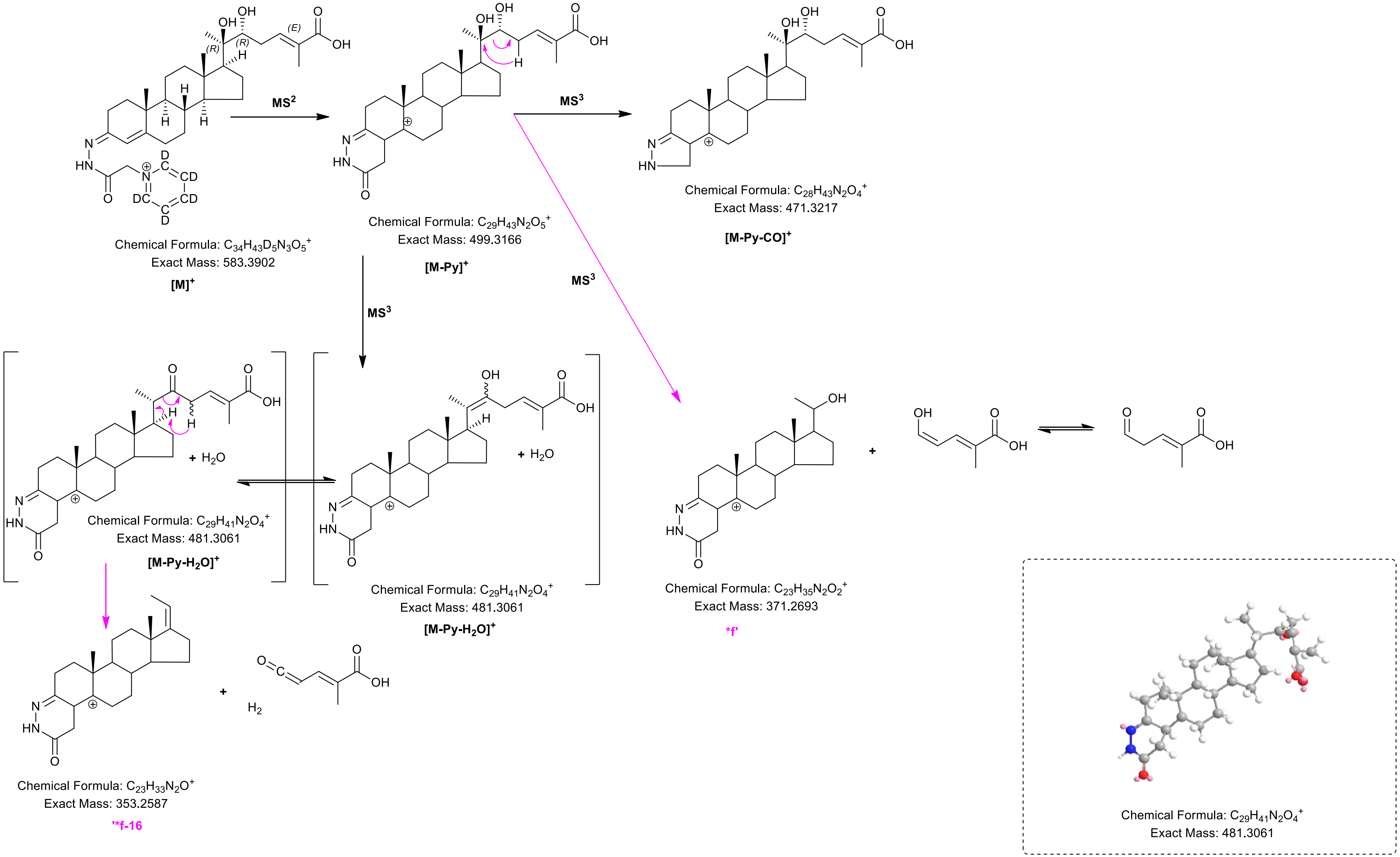
MS^2
MS^3
MS^3
MS^3
Chemical Formula: $C_{34}H_{43}D_5N_3O_5^+$
Exact Mass: 583.3902
[M]$^+$
Chemical Formula: $C_{29}H_{43}N_2O_5^+$
Exact Mass: 499.3166
[M-Py]$^+$
Chemical Formula: $C_{28}H_{43}N_2O_4^+$
Exact Mass: 471.3217
[M-Py-CO]$^+$
+ H_2O
Chemical Formula: $C_{29}H_{41}N_2O_4^+$
Exact Mass: 481.3061
[M-Py-H_2O]$^+$
+ H_2O
Chemical Formula: $C_{29}H_{41}N_2O_4^+$
Exact Mass: 481.3061
[M-Py-H_2O]$^+$
Chemical Formula: $C_{23}H_{35}N_2O_2^+$
Exact Mass: 371.2693
*f'
H_2
Chemical Formula: $C_{23}H_{33}N_2O^+$
Exact Mass: 353.2587
'*f-16
Chemical Formula: $C_{29}H_{41}N_2O_4^+$
Exact Mass: 481.3061

S3A

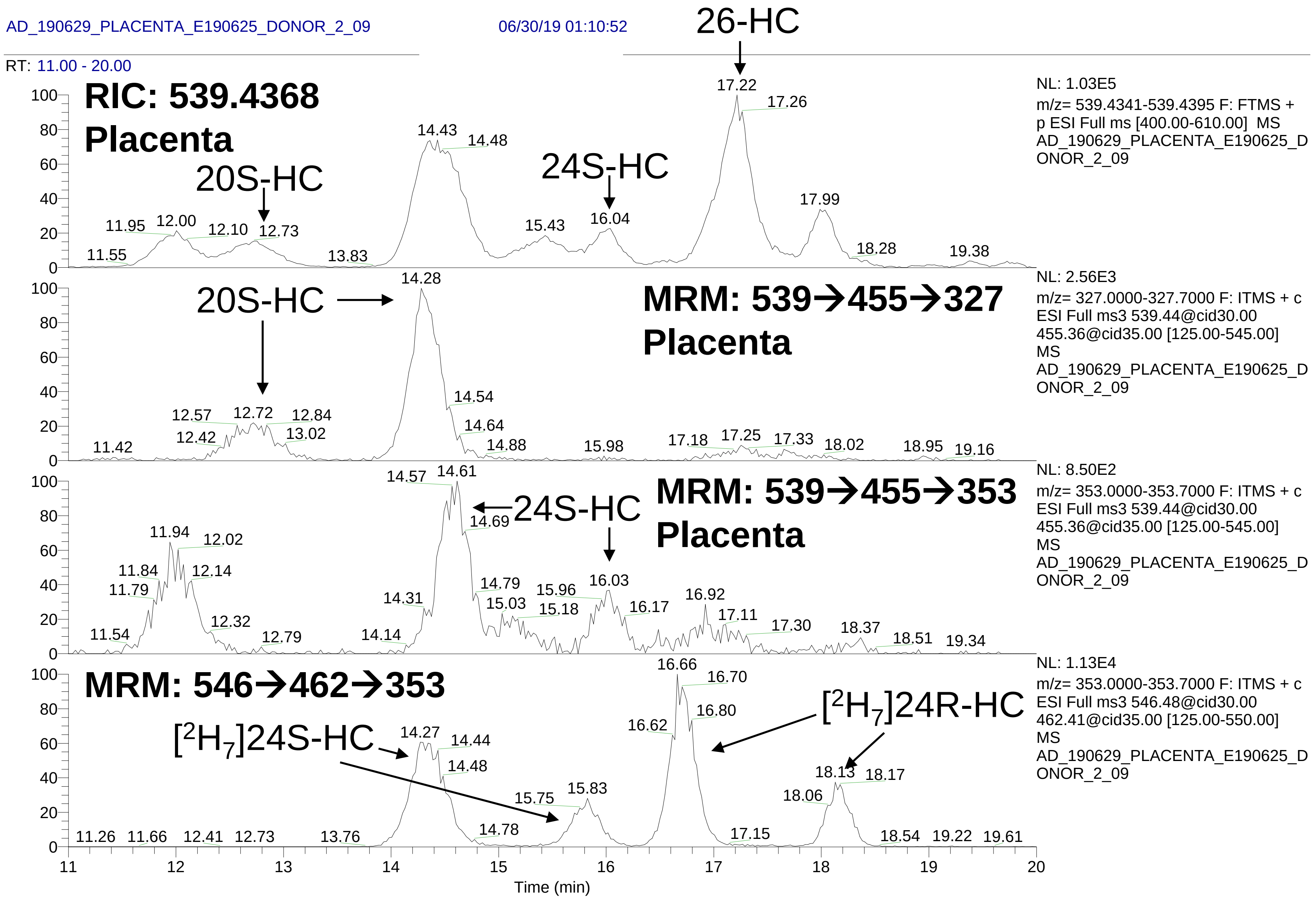
AD_190629_PLACENTA_E190625_DONOR_2_09
06/30/19 01:10:52
RT: 11.00 - 20.00
RIC: 539.4368
Placenta
20S-HC
24S-HC
26-HC
11.55
11.95
12.00
12.10
12.73
13.83
14.43
14.48
15.43
16.04
17.22
17.26
17.99
18.28
19.38
NL: 1.03E5
m/z= 539.4341-539.4395 F: FTMS + p ESI Full ms [400.00-610.00] MS AD_190629_PLACENTA_E190625_DONOR_2_09
MRM: 539→455→327
Placenta
20S-HC
14.28
11.42
12.57
12.42
12.72
12.84
13.02
14.54
14.64
14.88
15.98
17.18
17.25
17.33
18.02
18.95
19.16
NL: 2.56E3
m/z= 327.0000-327.7000 F: ITMS + c ESI Full ms3 539.44@cid30.00 455.36@cid35.00 [125.00-545.00] MS AD_190629_PLACENTA_E190625_DONOR_2_09
MRM: 539→455→353
Placenta
24S-HC
11.54
11.79
11.84
11.94
12.02
12.14
12.32
12.79
14.14
14.31
14.57
14.61
14.69
14.79
15.03
15.18
15.96
16.03
16.17
16.92
17.11
17.30
18.37
18.51
19.34
NL: 8.50E2
m/z= 353.0000-353.7000 F: ITMS + c ESI Full ms3 539.44@cid30.00 455.36@cid35.00 [125.00-545.00] MS AD_190629_PLACENTA_E190625_DONOR_2_09
MRM: 546→462→353
[2H7]24S-HC
[2H7]24R-HC
11.26
11.66
12.41
12.73
13.76
14.27
14.44
14.48
14.78
15.75
15.83
16.62
16.66
16.70
16.80
17.15
18.06
18.13
18.17
18.54
19.22
19.61
NL: 1.13E4
m/z= 353.0000-353.7000 F: ITMS + c ESI Full ms3 546.48@cid30.00 462.41@cid35.00 [125.00-550.00] MS AD_190629_PLACENTA_E190625_DONOR_2_09
0
20
40
60
80
100
11
12
13
14
15
16
17
18
19
20
Time (min)

S3B

AD_190629_PLACENTA_E190625_DONOR_2_09 06/30/19 01:10:52

AD_190629_PLACENTA_E190625_DONOR_ : 1 NL: 4.08E3
F: ITMS + c ESI Full ms3 539.44@cid30.00 45 .00]

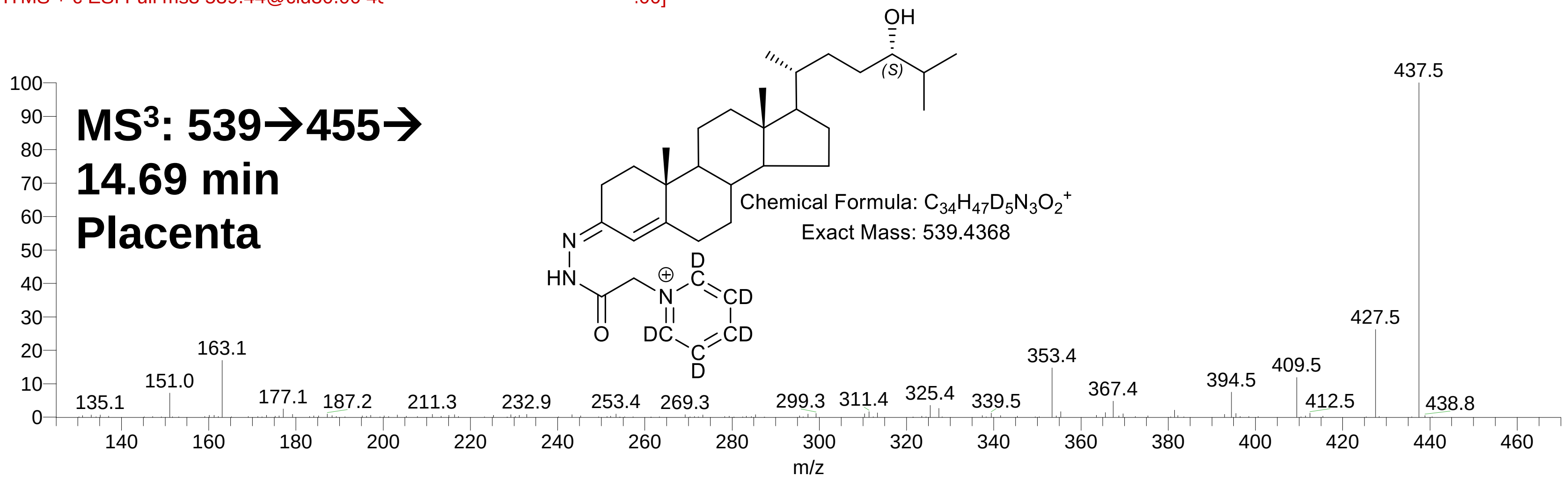

AD_190629_PLACENTA_E190625_DONOR_2_09 #3022 RT: 14.34 AV: 1 NL: 3.87E4
F: ITMS + c ESI Full ms3 546.48@cid30.00 462.41@cid35.00 [125.00-550.00]

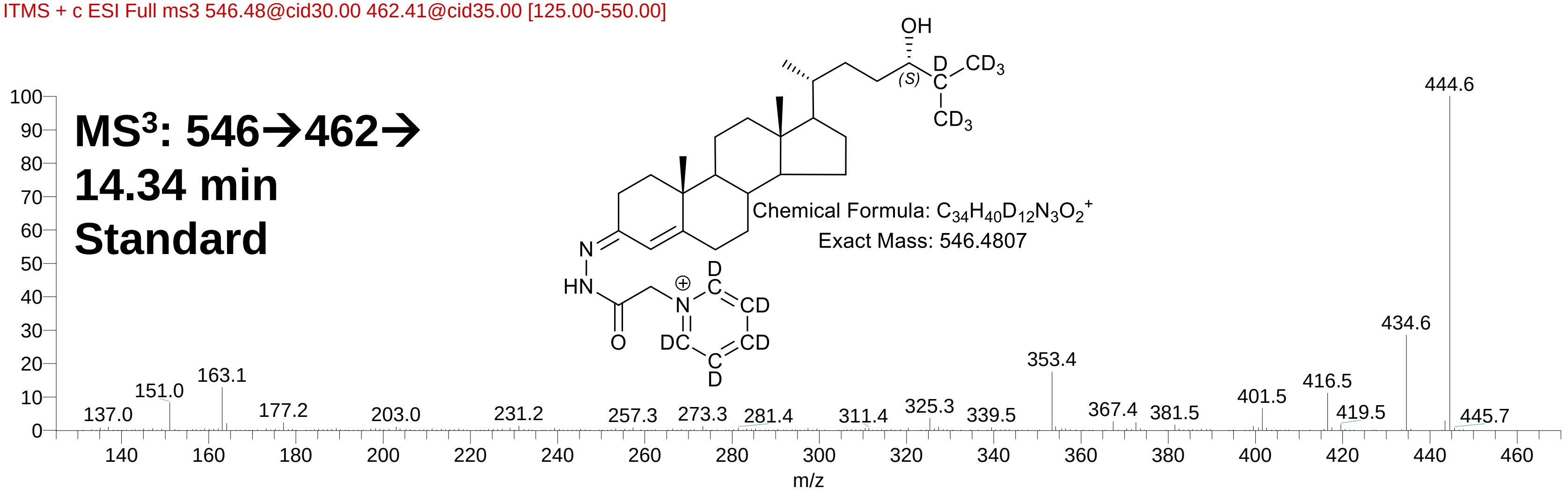

S3C

AD_190629_PLACENTA_E190625_DONOR_2_09 06/30/19 01:10:52

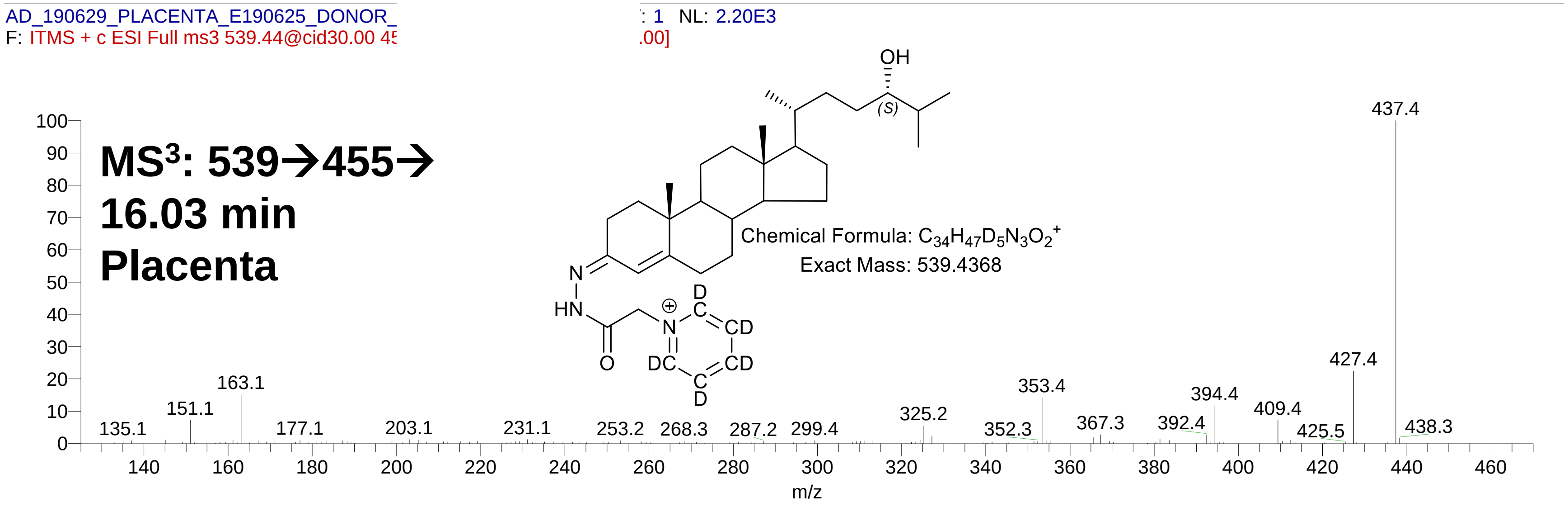

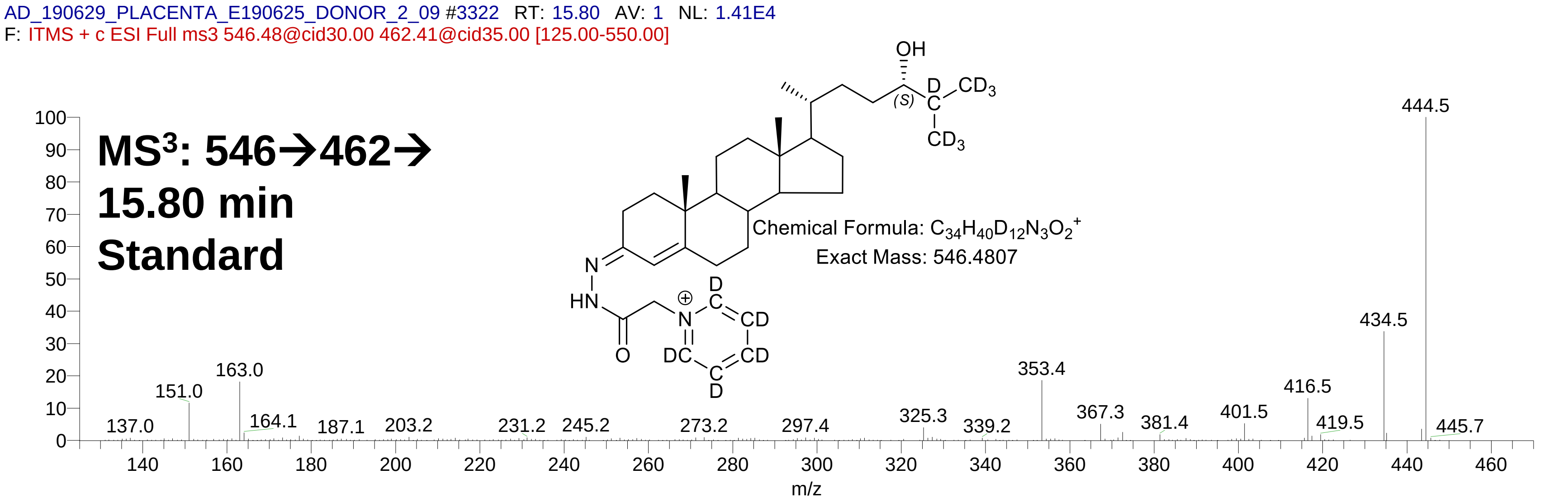

S3D

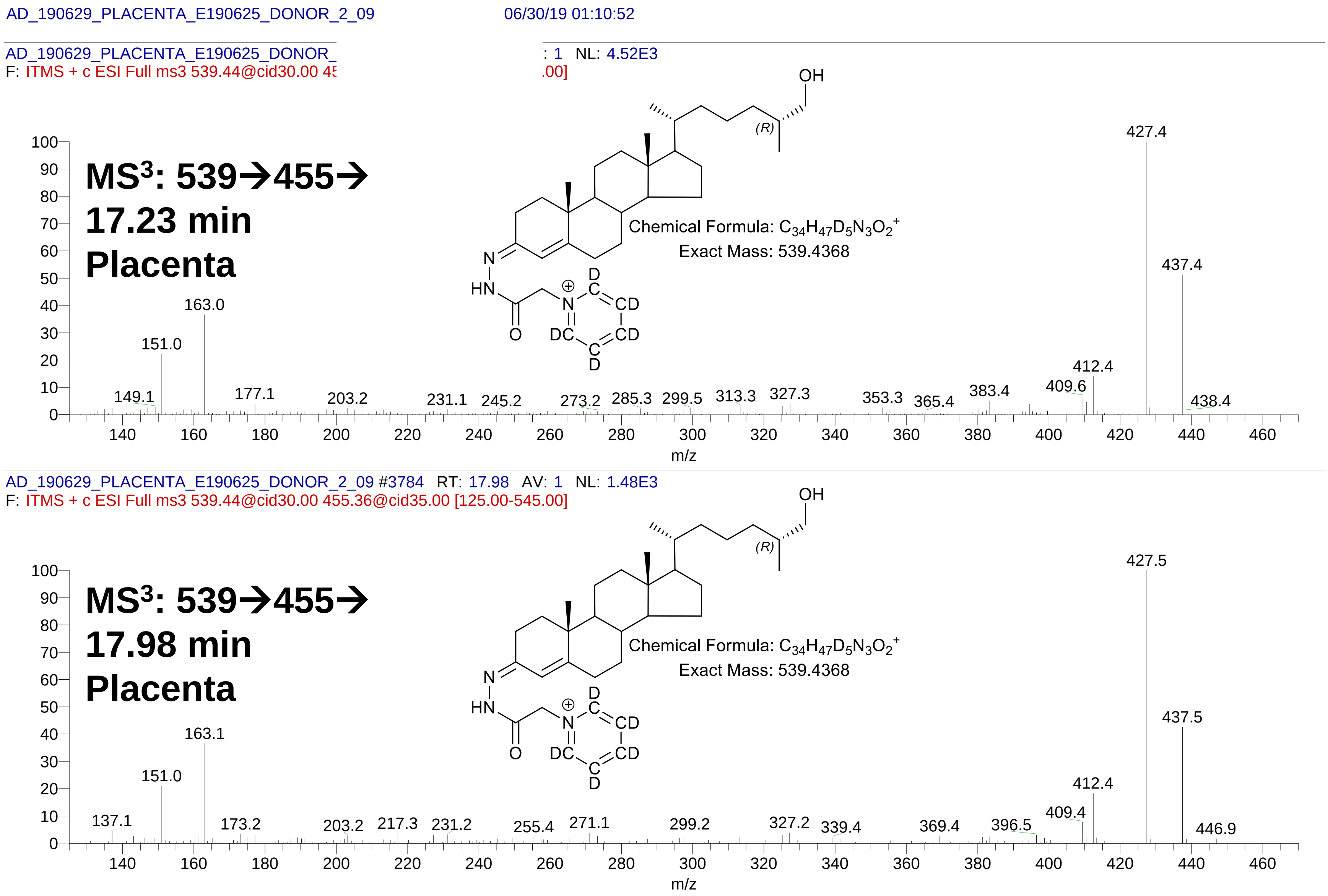

AD_190629_PLACENTA_E190625_DONOR_2_09
06/30/19 01:10:52
AD_190629_PLACENTA_E190625_DONOR_
: 1 NL: 4.52E3
F: ITMS + c ESI Full ms3 539.44@cid30.00 45
.00]
MS3: 539→455→
17.23 min
Placenta
Chemical Formula: C34H47D5N3O2+
Exact Mass: 539.4368
OH
(R)
N
HN
O
N
D
C
CD
CD
DC
C
D
427.4
437.4
412.4
409.6
438.4
383.4
365.4
353.3
327.3
313.3
299.5
285.3
273.2
245.2
231.1
203.2
177.1
163.0
151.0
149.1
m/z
AD_190629_PLACENTA_E190625_DONOR_2_09 #3784 RT: 17.98 AV: 1 NL: 1.48E3
F: ITMS + c ESI Full ms3 539.44@cid30.00 455.36@cid35.00 [125.00-545.00]
MS3: 539→455→
17.98 min
Placenta
Chemical Formula: C34H47D5N3O2+
Exact Mass: 539.4368
427.5
437.5
412.4
409.4
446.9
396.5
369.4
339.4
327.2
299.2
271.1
255.4
231.2
217.3
203.2
173.2
163.1
151.0
137.1
m/z

S3E

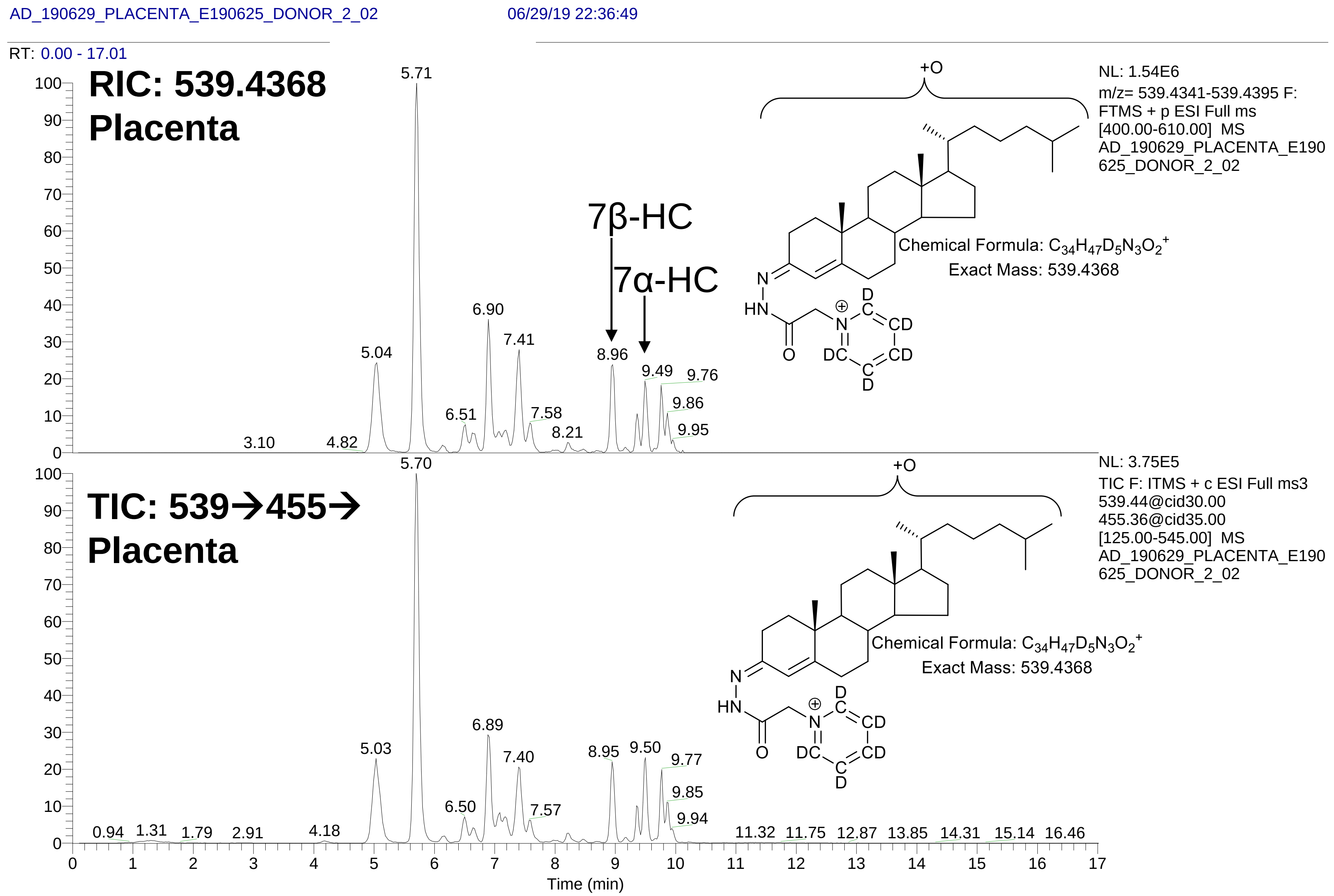
AD_190629_PLACENTA_E190625_DONOR_2_02
06/29/19 22:36:49
RT: 0.00 - 17.01
RIC: 539.4368
Placenta
TIC: 539→455→
Placenta
7β-HC
7α-HC
+O
NL: 1.54E6
m/z= 539.4341-539.4395 F: FTMS + p ESI Full ms [400.00-610.00] MS AD_190629_PLACENTA_E190625_DONOR_2_02
NL: 3.75E5
TIC F: ITMS + c ESI Full ms3 539.44@cid30.00 455.36@cid35.00 [125.00-545.00] MS AD_190629_PLACENTA_E190625_DONOR_2_02
Chemical Formula: $C_{34}H_{47}D_5N_3O_2^+$
Exact Mass: 539.4368
Time (min)

S3F

AD_190629_PLACENTA_E190625_DONOR_2_02 06/29/19 22:36:49

AD_190629_PLACENTA_E190625_DONOR_2_02 #1979 RT: 8.95 AV: 1
F: ITMS + c ESI Full ms3 539.44@cid30.00 455.36@cid35.00 [125.00-545.0

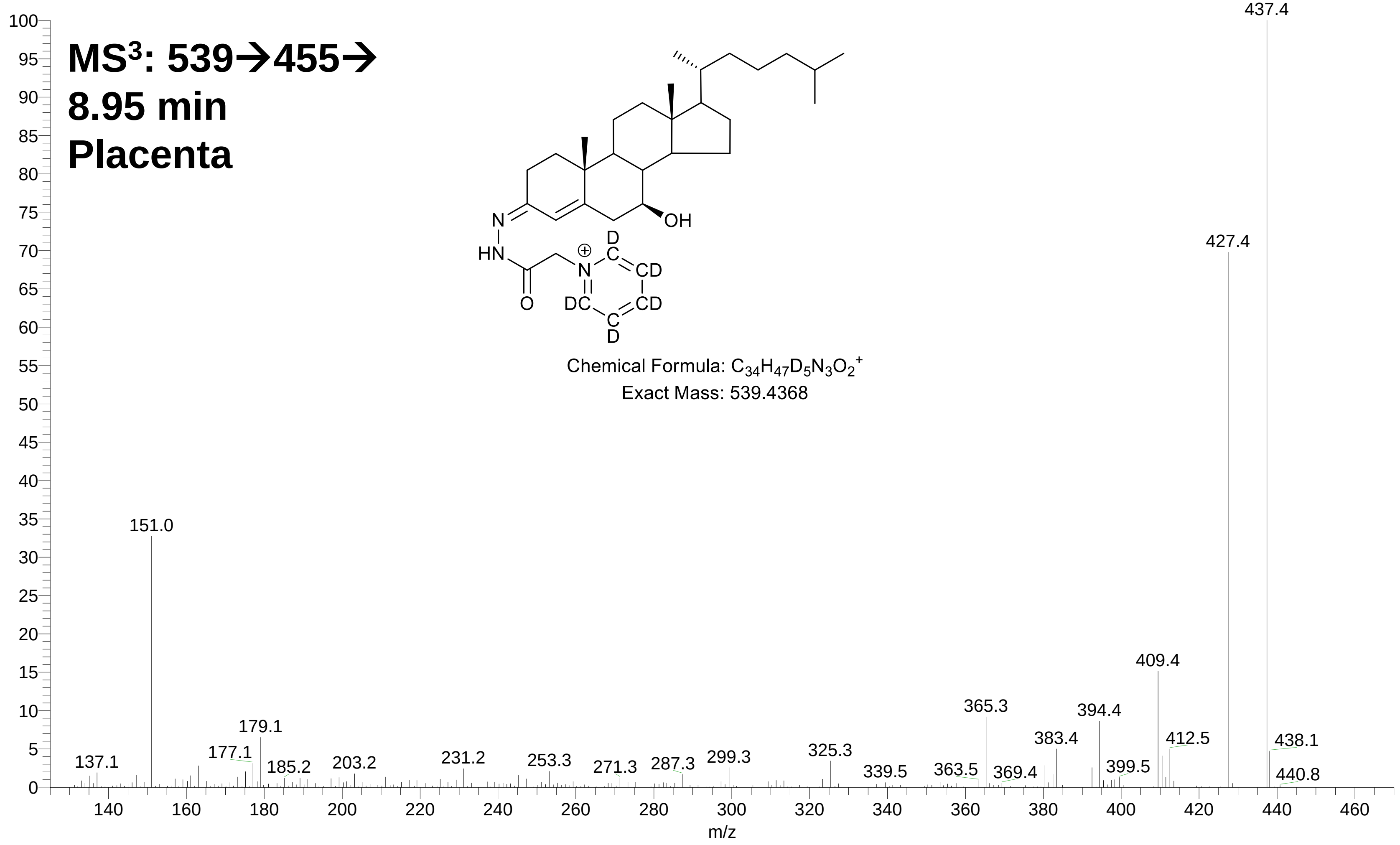

S3G

AD_190629_PLACENTA_E190625_DONOR_2_02 06/29/19 22:36:49

AD_190629_PLACENTA_E190625_DONOR_2_02 #2104 RT: 9.48 AV: 1
F: ITMS + c ESI Full ms3 539.44@cid30.00 455.36@cid35.00 [125.00-545.0

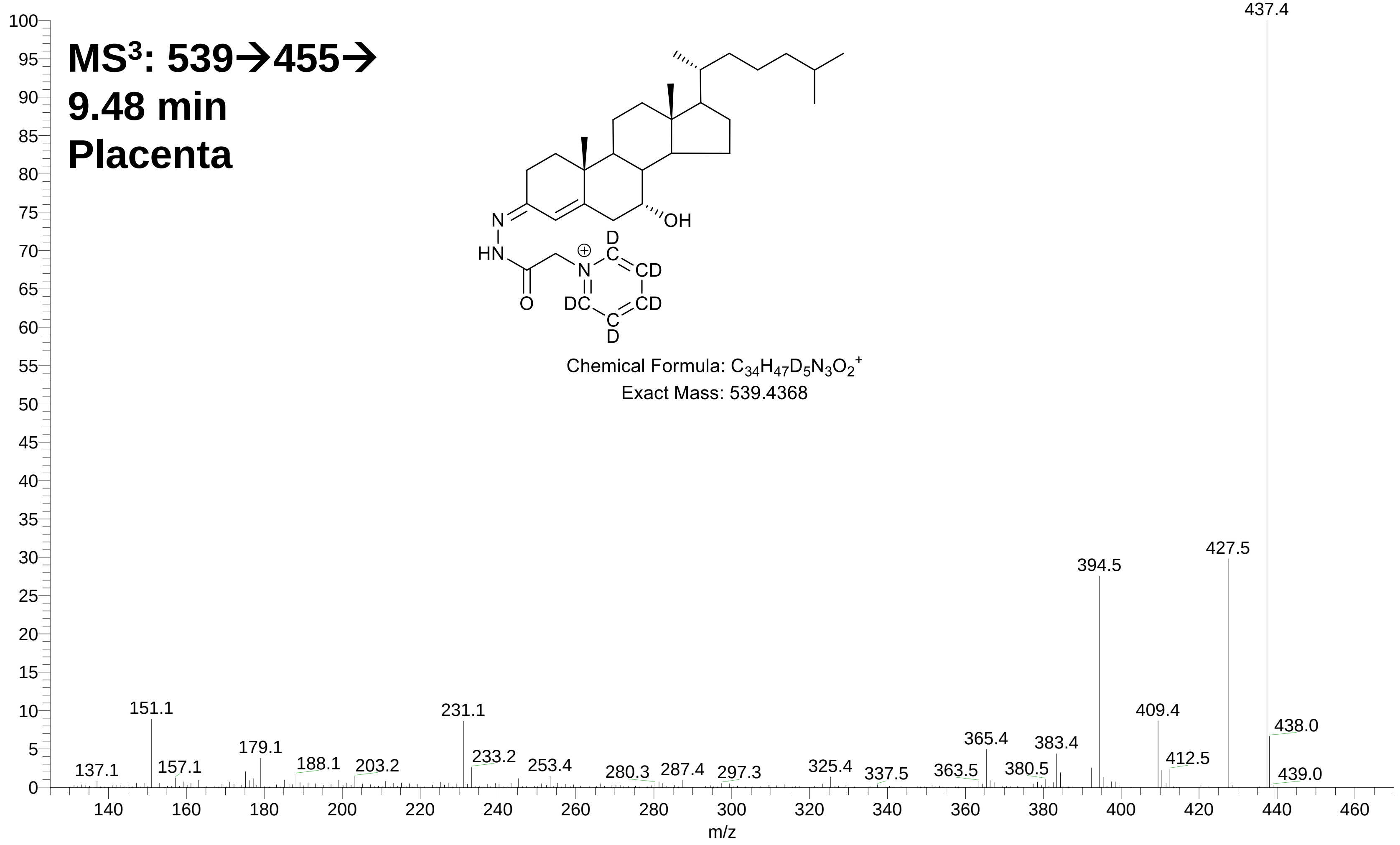

S4A

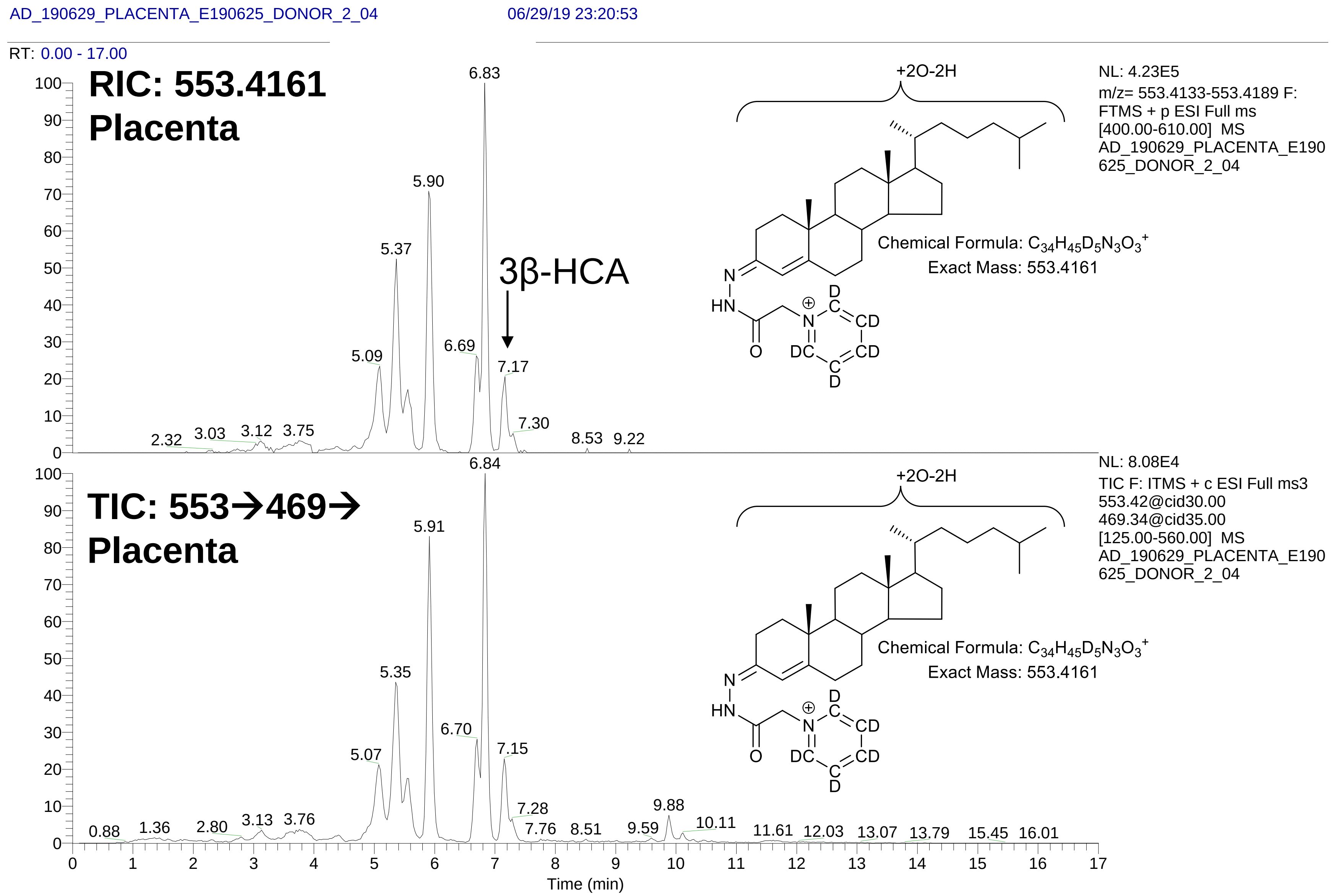
AD_190629_PLACENTA_E190625_DONOR_2_04
06/29/19 23:20:53
RT: 0.00 - 17.00
RIC: 553.4161
Placenta
6.83
5.90
5.37
5.09
6.69
7.17
7.30
2.32
3.03
3.12
3.75
8.53
9.22
3β-HCA
+2O-2H
NL: 4.23E5
m/z= 553.4133-553.4189 F: FTMS + p ESI Full ms [400.00-610.00] MS AD_190629_PLACENTA_E190625_DONOR_2_04
Chemical Formula: $C_{34}H_{45}D_5N_3O_3^+$
Exact Mass: 553.4161
TIC: 553→469→
Placenta
6.84
5.91
5.35
5.07
6.70
7.15
7.28
7.76
8.51
9.59
9.88
10.11
11.61
12.03
13.07
13.79
15.45
16.01
0.88
1.36
2.80
3.13
3.76
NL: 8.08E4
TIC F: ITMS + c ESI Full ms3 553.42@cid30.00 469.34@cid35.00 [125.00-560.00] MS AD_190629_PLACENTA_E190625_DONOR_2_04
Time (min)

S4B

AD_190629_PLACENTA_E190625_DONOR_2_04 06/29/19 23:20:53

AD_190629_PLACENTA_E190625_DONOR_2_04 #1540 RT: 7.15 AV:
F: ITMS + c ESI Full ms3 553.42@cid30.00 469.34@cid35.00 [125.00-560

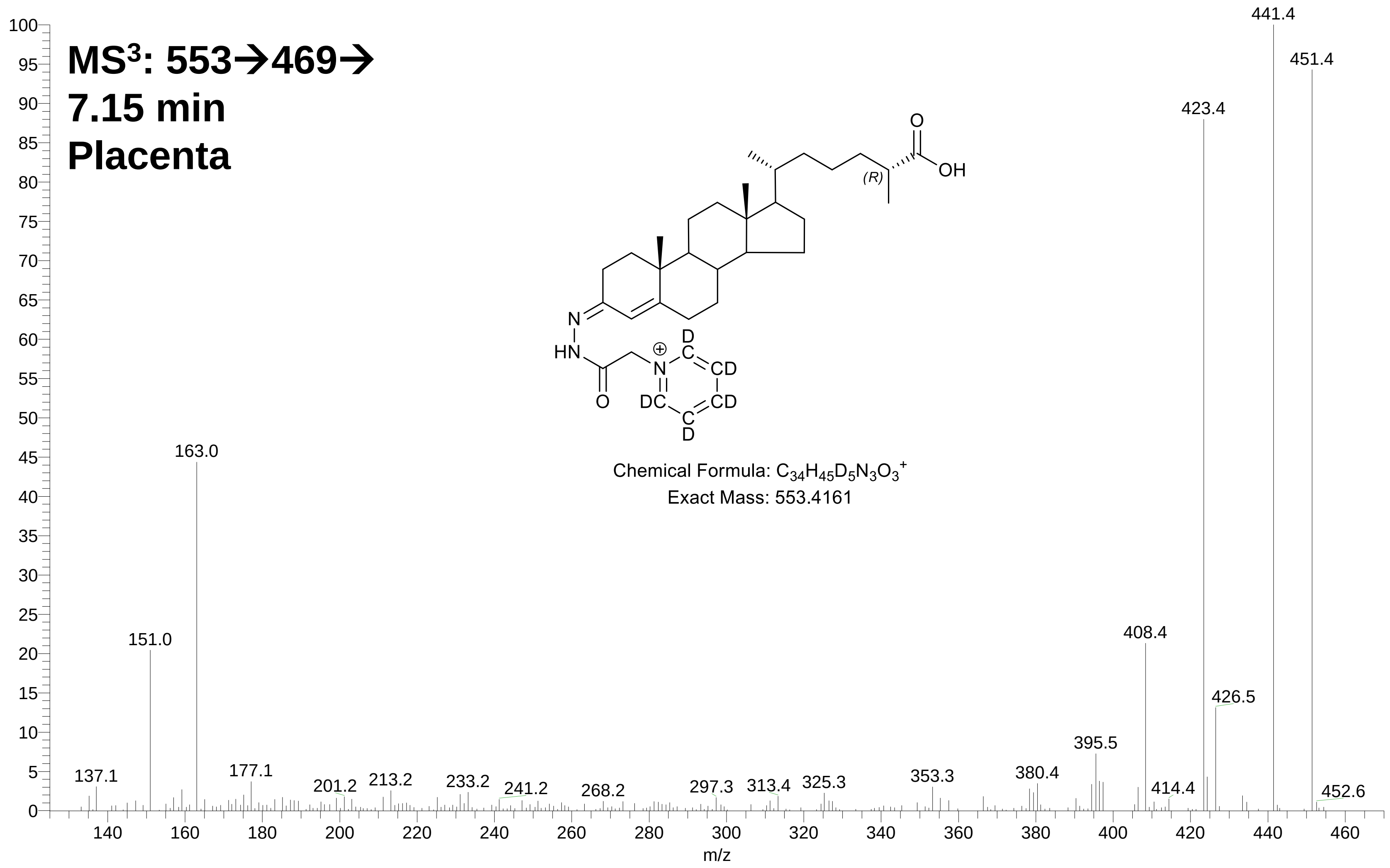

S5A

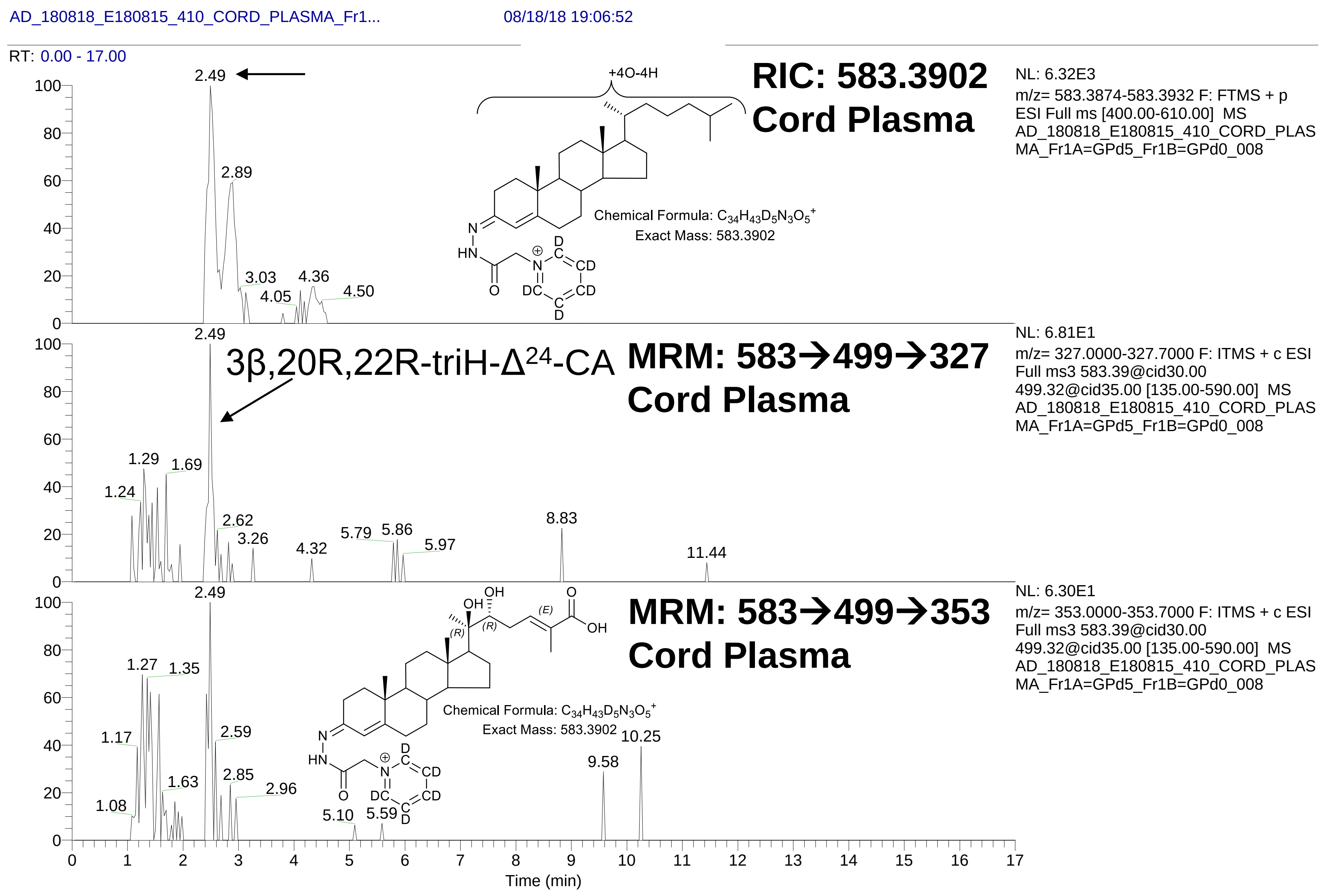

AD_180818_E180815_410_CORD_PLASMA_Fr1...
08/18/18 19:06:52
RT: 0.00 - 17.00
RIC: 583.3902
Cord Plasma
NL: 6.32E3
m/z= 583.3874-583.3932 F: FTMS + p ESI Full ms [400.00-610.00] MS AD_180818_E180815_410_CORD_PLASMA_Fr1A=GPd5_Fr1B=GPd0_008
+4O-4H
Chemical Formula: C34H43D5N3O5+
Exact Mass: 583.3902
2.49
2.89
3.03
4.05
4.36
4.50
3β,20R,22R-triH-Δ24-CA
MRM: 583→499→327
Cord Plasma
NL: 6.81E1
m/z= 327.0000-327.7000 F: ITMS + c ESI Full ms3 583.39@cid30.00 499.32@cid35.00 [135.00-590.00] MS AD_180818_E180815_410_CORD_PLASMA_Fr1A=GPd5_Fr1B=GPd0_008
1.24
1.29
1.69
2.62
3.26
4.32
5.79
5.86
5.97
8.83
11.44
MRM: 583→499→353
Cord Plasma
NL: 6.30E1
m/z= 353.0000-353.7000 F: ITMS + c ESI Full ms3 583.39@cid30.00 499.32@cid35.00 [135.00-590.00] MS AD_180818_E180815_410_CORD_PLASMA_Fr1A=GPd5_Fr1B=GPd0_008
Chemical Formula: C34H43D5N3O5+
Exact Mass: 583.3902
1.08
1.17
1.27
1.35
1.63
2.59
2.85
2.96
5.10
5.59
9.58
10.25
Time (min)

S5B

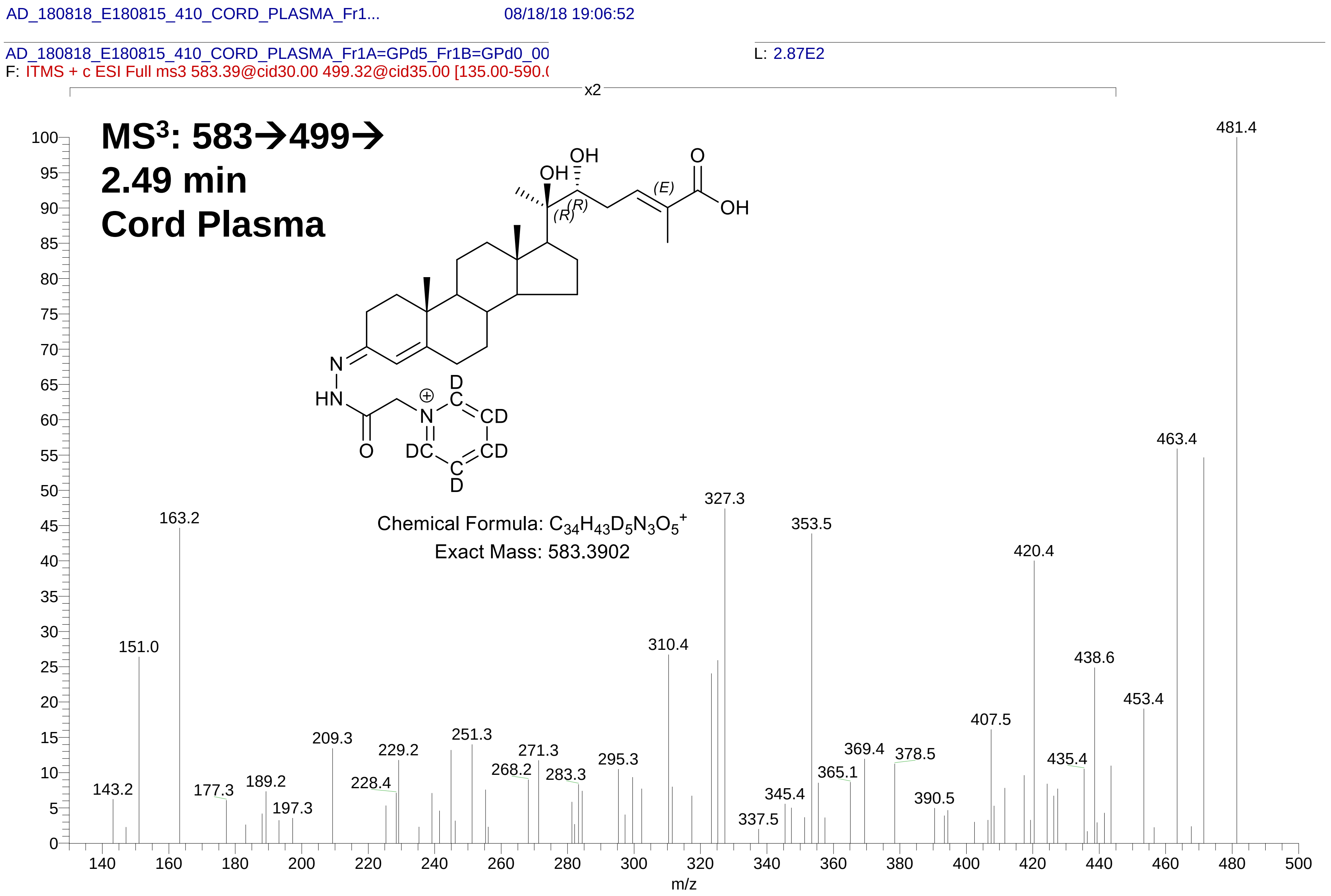
AD_180818_E180815_410_CORD_PLASMA_Fr1...
08/18/18 19:06:52
AD_180818_E180815_410_CORD_PLASMA_Fr1A=GPd5_Fr1B=GPd0_00
F: ITMS + c ESI Full ms3 583.39@cid30.00 499.32@cid35.00 [135.00-590.(
L: 2.87E2
x2
MS³: 583→499→
2.49 min
Cord Plasma
OH
OH
O
OH
(E)
(R)
(R)
N
HN
O
D
C
CD
N
DC
CD
C
D
Chemical Formula: C34H43D5N3O5+
Exact Mass: 583.3902
481.4
463.4
327.3
353.5
163.2
420.4
151.0
310.4
438.6
453.4
407.5
209.3
251.3
229.2
271.3
369.4
378.5
435.4
295.3
268.2
365.1
283.3
143.2
189.2
177.3
228.4
345.4
197.3
390.5
337.5
m/z
0
5
10
15
20
25
30
35
40
45
50
55
60
65
70
75
80
85
90
95
100
140
160
180
200
220
240
260
280
300
320
340
360
380
400
420
440
460
480
500

S5C

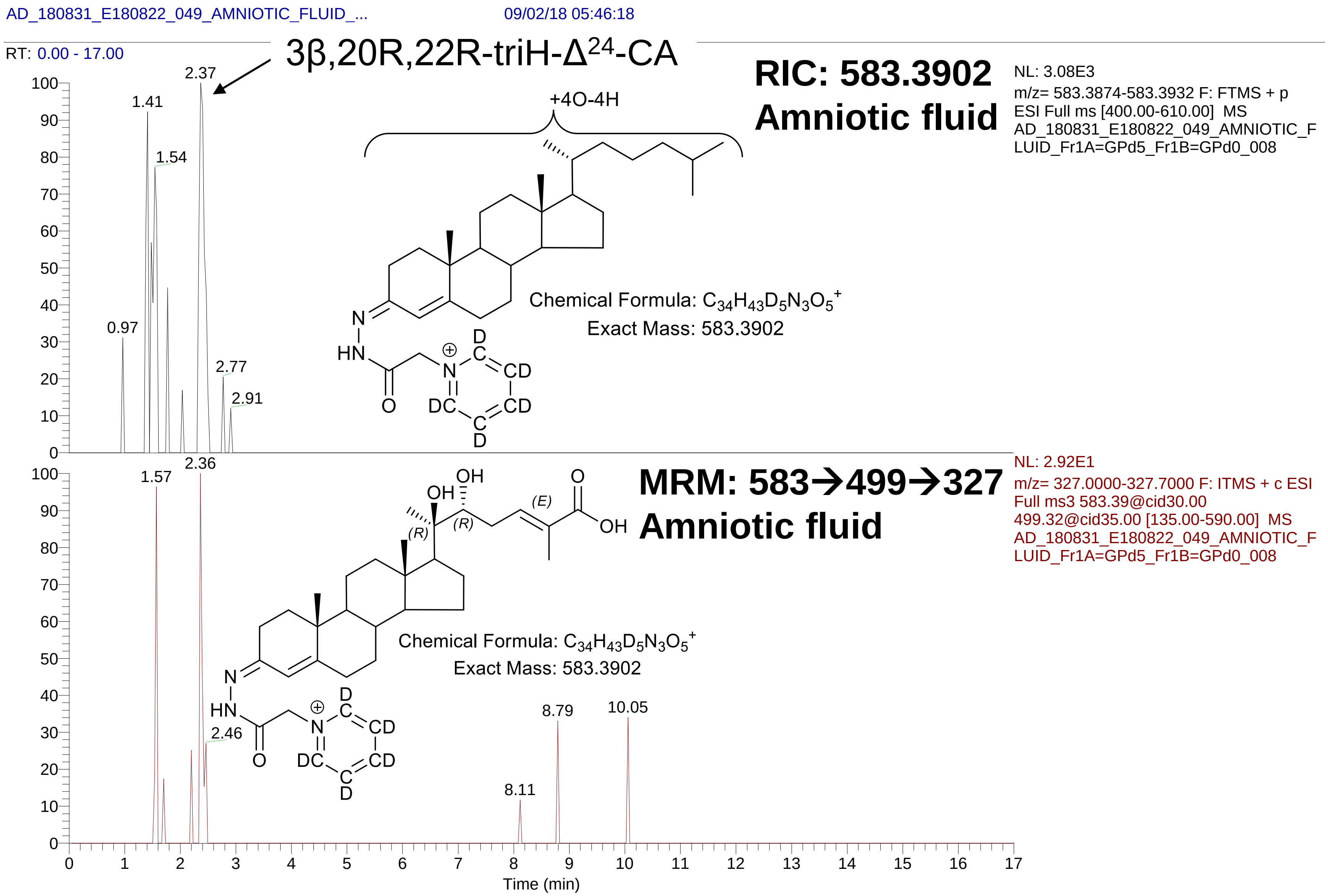

AD_180831_E180822_049_AMNIOTIC_FLUID_...
09/02/18 05:46:18
RT: 0.00 - 17.00
3β,20R,22R-triH-Δ24-CA
RIC: 583.3902
Amniotic fluid
NL: 3.08E3
m/z= 583.3874-583.3932 F: FTMS + p ESI Full ms [400.00-610.00] MS AD_180831_E180822_049_AMNIOTIC_FLUID_Fr1A=GPd5_Fr1B=GPd0_008
+4O-4H
Chemical Formula: C34H43D5N3O5+
Exact Mass: 583.3902
2.37
1.41
1.54
0.97
2.77
2.91
MRM: 583→499→327
Amniotic fluid
NL: 2.92E1
m/z= 327.0000-327.7000 F: ITMS + c ESI Full ms3 583.39@cid30.00 499.32@cid35.00 [135.00-590.00] MS AD_180831_E180822_049_AMNIOTIC_FLUID_Fr1A=GPd5_Fr1B=GPd0_008
Chemical Formula: C34H43D5N3O5+
Exact Mass: 583.3902
2.36
1.57
2.46
8.79
10.05
8.11
Time (min)

S5D

AD_180831_E180822_049_AMNIOTIC_FLUID_... 09/02/18 05:46:18

AD_180831_E180822_049_AMNIOTIC_FLUID_Fr1A=GPd5_Fr1B=GPd0_008 AV: 2 NL: 8.68E1
F: ITMS + c ESI Full ms3 583.39@cid30.00 499.32@cid35.00 [135.00-590.00]

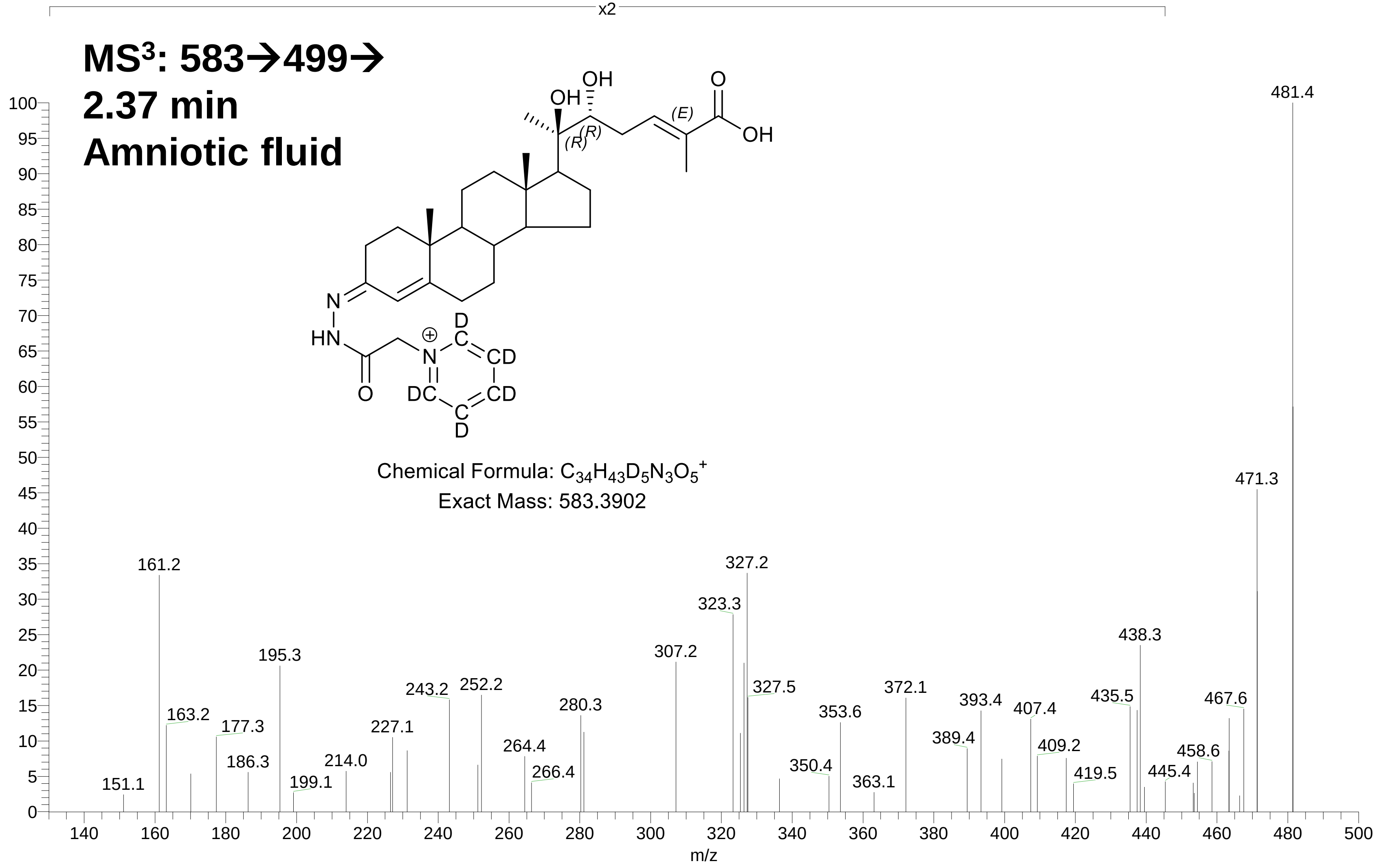

Supplement: Supplementary file 1 [file DataSheet_1.pdf]
